# Rapid Sequence Identification of Foot-and-Mouth Disease Virus Utilizing FMDV-ONTAPS: The Oxford Nanopore Technologies Amplicon P1 Sequencing Protocol

**DOI:** 10.3390/v18040418

**Published:** 2026-03-28

**Authors:** Sean Yeo, Kate Hole, Taeyo Chestley, Grace E. Seo, Anna Majer, Katherine Handel, Michelle Nebroski, Oliver Lung, Charles Nfon, Shawn Babiuk

**Affiliations:** 1 National Centre for Foreign Animal Diseases, Canadian Food Inspection Agency, 1015 Arlington Street, Winnipeg, MB R3E 3R2, Canada; sean.yeo@inspection.gc.ca (S.Y.); kate.hole@inspection.gc.ca (K.H.); katherine.handel@inspection.gc.ca (K.H.); michelle.nebroski@inspection.gc.ca (M.N.); oliver.lung@inspection.gc.ca (O.L.); charles.nfon@inspection.gc.ca (C.N.); 2 National Microbiology Laboratory Branch, Public Health Agency of Canada, 1015 Arlington Street, Winnipeg, MB R3E 3R2, Canada; taeyo.chestley@phac-aspc.gc.ca (T.C.); grace.seo@phac-aspc.gc.ca (G.E.S.); anna.majer@phac-aspc.gc.ca (A.M.); 3 Department of Medical Microbiology and Infectious Diseases, Max Rady College of Medicine, University of Manitoba, 727 McDermot Avenue, Winnipeg, MB R3E 3P5, Canada

**Keywords:** FMDV, FMDV serotyping, Nanopore sequencing, MinION Mk1D, flongle, FMDV-ONTAPS

## Abstract

Diagnostic testing of foot-and-mouth disease virus (FMDV) currently utilizes reverse transcription quantitative PCR (RT-qPCR) to detect the presence of viral RNA and double antibody sandwich ELISAs (DAS-ELISAs) to determine viral serotype. Serotype identification is critical to support informed vaccine selection to combat outbreaks. While DAS-ELISAs are capable of serotype identification, the test suffers from low sensitivity and requires a viral isolate for successful detection. In this study, we developed FMDV-ONTAPS: an Oxford Nanopore Technologies Amplicon P1 Sequencing protocol involving reverse transcription-PCR to amplify P1 of the FMDV genome, and Nanopore sequencing of the amplicons to provide genetic data for serotype and subtype/topotype identification. FMDV isolates representing all seven serotypes were successfully sequenced with this method. Additionally, the protocol successfully provided serotype identification from a variety of specimen matrices obtained from experimentally infected animals that included milk, serum, oral and nasal swabs, tissue suspensions, vesicular fluid, and oral fluid. The limit of detection for FMDV cell culture isolates was comparable for both sequencing and RT-qPCR detection. RT-qPCR Cq values for clinical samples evaluated ranged from 8 to 28.21. Sequencing was successful for all samples except for a single tissue suspension sample (Cq of 28.21). Identification of FMDV serotype in clinical samples is critical for effective outbreak response, and Nanopore sequencing offers a timelier and more sensitive alternative to DAS-ELISAs.

## 1. Introduction

Foot-and-mouth disease (FMD) has broad host tropism for cloven-hoofed animals, including the key livestock species, cattle, sheep, goats, and swine. The causal agent of FMD is the FMD virus (FMDV), which belongs to the family *Picornaviridae* under the genus *Aphthovirus* [1]. FMDV has seven distinct serotypes–O, A, C, Asia-1, and Southern African Territories (SAT)-1, SAT-2, and SAT-3, which are antigenically variable [1]. The geographic distribution of FMDV occurs in seven geographic pools with an uneven distribution of serotypes and strains [2]. FMD continues to impact the animal health industry, where the virus is endemic and is a constant threat to countries that are free of the disease. Countries where outbreaks occur lose their World Organization of Animal Health (WOAH) status as FMD-free without vaccination and are barred from exporting animals or animal products [3,4]. This can have significant financial consequences for countries that rely on livestock exports. Furthermore, FMDV has a significant impact on animals’ health. Clinical manifestations of FMD include fever, shivering, profuse salivation, and the characteristic formation of vesicles on the mouth and feet [5]. These painful lesions limit the animal’s ability to eat, drink, or walk and can subsequently lead to limited weight gain, decreased milk production, and loss of draught power [4]. FMD typically has more severe effects in neonates, with reported mortality rates as high as 94% in lambs, 80% in calves, and 100% in suckling piglets [6]. Typical clinical signs are not exclusive to FMD; therefore, distinguishing the disease from other vesicular diseases is reliant on differential diagnostic testing [7].

The rapid diagnosis of a vesicular disease is critical to identify and differentiate FMDV from swine vesicular disease virus (SVDV), vesicular stomatitis virus (VSV), and senecavirus A (SVA) [8]. Once FMDV has been diagnosed in the laboratory using a specific real-time reverse transcription quantitative PCR (RT-qPCR) assay, there is a further requirement to identify the immunologically distinct serotype (O, A, C, Asia 1, SAT 1, SAT 2, and SAT 3) along with the associated subtype/topotype and lineage. Vaccination against one serotype of FMDV does not necessarily confer protection against the other serotypes [9]. Therefore, serotype identification is crucial for outbreak response and the informed selection of the appropriate vaccine for disease control.

The current and most commonly used diagnostic method for identifying FMDV serotypes is the Double Antibody Sandwich ELISA (DAS-ELISA). However, the use of polyclonal antibodies in this assay often results in low specificity due to antigenic cross-reactivity. Another major pitfall of the DAS-ELISA is the woefully low sensitivity of 80–90% for positive bovine samples and <80% for porcine samples [5,10,11,12]. Due to these issues, generation of a viral isolate is often required to provide adequate antigen for accurate serotype classification, and this prerequisite significantly prolongs test turnaround times.

Due to the genetic diversity of the virus, attempts to produce and validate an RT-qPCR assay capable of distinguishing FMDV serotypes have fallen short when testing across multiple FMDV pools. Current TaqMan probe-based RT-qPCR serotyping assays are all geographically restricted to distinguish serotypes in specific regions. For example, Jamal and Belsham designed primer/probe sets capable of distinguishing FMDV serotypes O, A, and Asia 1 but only for subtypes circulating in the Middle East [13]. Likewise, Bachanek-Bankowska et al. were able to discern FMDV O, A, SAT 1, and SAT 2 but were restricted to viruses found in East Africa [14]. Several other assays have been reported, but all suffer from the geographic restriction caveat [15,16,17,18,19]. Unfortunately, these current assays have limited utility if the need to serotype an unknown or new isolate of FMDV arises. The isolated FMDV genome used in the preliminary identification of the virus could be sequenced using Sanger sequencing of the VP1 region to provide serotype identification [20]. However, this protocol requires knowledge of the suspected serotype/strain to choose the correct primer pairs for VP1 amplification. Without this information, identifying the serotype would be a complex and lengthy process.

Next Generation Sequencing (NGS) can be used as an alternative to Sanger sequencing, but it typically requires a large initial investment. Thus far, Nanopore sequencing is one of the most cost-effective sequencing technologies, with estimated costs of approximately $21–42 USD per Gigabase (Gb) with the PromethION versus Illumina at $50–63 USD/Gb with the NovaSeq 550, and Sanger at a staggering cost of $13,000 USD/Gb [21]. Oxford Nanopore Technologies (ONT) offers the MinION Mk1D platform at a low cost of entry in comparison to other sequencing devices. The Mk1D platform is compact enough to fit in the palm of a hand and is powered through a USB-C port of a laptop. The MinION software, MinKNOW (v24.06.8 and newer, https://nanoporetech.com/software/devices/minion-mk1d/software, accessed on 14 May 2023), is a user-friendly interface that does not require extensive bioinformatics knowledge or command line expertise. One advantage of Nanopore sequencing over other methods is the rapid processing times. Several groups have reported a 5–6 h turnaround time with Nanopore sequencing from sample receipt to final sequence analysis for virus identification [22,23,24,25,26]. This is as opposed to two days with Sanger, and up to a week for other NGS methods [27]. The cost of Nanopore sequencing has reduced with the advent of the Flongle in 2019. The Flongle is an adapter for Nanopore sequencing platforms that allows the use of smaller single-use flow cells priced at approximately $80 USD–about a tenth of the cost of a MinION flow cell [27]. This equates to an overall cost per Gb of approximately $15 USD (excluding the cost of the MinION Mk1D and computer).

Given the extensive and ever-growing genetic diversity of FMDV, the sensitivity issues of the DAS-ELISA, and the challenges of producing a non-restricted RT-qPCR serotyping assay, Nanopore sequencing provides a solution for rapid serotyping of FMDV. This methodology has been preliminarily explored with success, producing sequence data from both full and partial FMDV genome sequences from limited and ideal sample sets [24,28]. In this study, we developed a protocol, FMDV Oxford Nanopore Technologies Amplicon P1 Sequencing (FMDV-ONTAPS), which can rapidly serotype FMDV from RT-qPCR positive samples by amplifying the P1 region of the FMDV genome, followed by sequence generation using the ONT MinION Mk1D portable sequencer. To further reduce cost and increase accessibility, we utilized the Flongle adapter and the compatible flow cells. The sequenced P1 region was then mapped against reference sequences to generate a consensus sequence used for serotype identification, as well as providing topotype and lineage information.

## 2. Materials and Methods

### 2.1. Sample Preparation

Representative cell culture isolates from all seven FMDV serotypes (Table 1) were tested to determine the validity of the results and were used to assess the assay limit of detection (LOD). Analytical specificity of the assay was evaluated using a panel of non-target, differential viruses. Clinical samples from various sample matrices, including milk, serum, swabs (oral and nasal), vesicular fluid, oral fluids, and various tissue types from experimentally infected animals, were tested. All clinical samples were obtained as remnants from previous animal experiments approved by the Canadian Science Center for Human and Animal Health Animal Care Committee. Animal use document numbers are as follows: C-11-003, C-14-002, C-16-008, C-16-010, C-18-002, and C-18-004.

Tissue samples were prepared as previously described [11]. Briefly, tissues were homogenized in Dulbecco’s Phosphate Buffered Saline (D-PBS) to produce 10% weight/volume suspensions. These suspensions were then clarified by centrifugation (2000× *g* for 20 minutes (mins) at 4 °C) prior to RNA extraction. Swabs (oral, nasal) were stored in BD Universal Viral Transport Media (Becton, Dickinson, and Company, Mississauga, ON, Canada) and vortexed to release the virus prior to RNA extraction. Oral fluids were clarified by centrifugation using the same conditions as clarification of tissue homogenates. All other sample types were used directly for RNA extraction. To assess the LOD of Nanopore sequencing, ten-fold serial dilutions of the stock virus were tested in duplicate by a second analyst. For mixtures of FMDV serotypes or strains, extracted RNA from isolates was combined at a 1:1 ratio. The same was done for mixtures of FMDV and other non-target vesicular disease viruses.

### 2.2. FMDV DAS-ELISA

The classical DAS-ELISAs were conducted as described previously [1,11].

### 2.3. RNA Extraction and Real-Time RT-PCR

RNA extraction on all sample types was performed using the Applied Biosystems MagMax-96 Viral RNA Isolation Kit (AMB18365, ThermoFisher Scientific, Burlington, ON, Canada), following the manufacturer’s protocol. RNA was extracted using a KingFisher APEX (5400930, ThermoFisher Scientific) and then assessed for FMDV positivity by RT-qPCR as described previously [29]. Briefly, template RNA (5 μL) was added to a 20 μL mastermix comprising TaqMan^®^ Fast Virus 1-Step master mix (4444436, ThermoFisher Scientific), 0.5 μM each of the forward and reverse primers and 0.2 μM probe. RT-qPCR was performed on a QuantStudio™ 7 Pro (A43162, ThermoFisher Scientific) machine using the cycling conditions: 50 °C for 5 mins, 95 °C for 20 seconds (s), followed by 40 cycles of 95 °C for 15 s and 60 °C for 45 s. A cut-off quantification cycle value (Cq) of ≤35.99 was considered positive for the presence of FMDV genome in the sample.

### 2.4. cDNA Synthesis and PCR

Targeted Reverse Transcription (RT) of FMDV genomic RNA was performed using SuperScript™ IV First-Strand Synthesis System (18091050, ThermoFisher Scientific) as per the manufacturer’s instructions, using the primer Rev 6 (5′–GGC GGC CGC TTT TTT TTT TTT TTT–3′) (LGC Biosearch Technologies, Teddington, UK) at a concentration of 2 µM [30].

The cDNA template was then used to generate FMDV P1 PCR amplicons with universal FMDV primers described previously by Xu et al. [31]. Briefly, 5 µL of the cDNA template was added to reactions comprising 25 µL of 2× Platinum SuperFi II PCR Master Mix (12368010, ThermoFisher Scientific), 15 µL of nuclease-free water and 2.5 µL of each universal FMDV primer at a final concentration of 0.5 µM (Univ F: 5′–TGGTGACAGGCTAAGGATG–3′ and Univ R: 5′–GCCCRGGGTTGGACTC–3′) (LGC Biosearch Technologies) [31]. The cycling conditions consisted of an initial denaturation step at 98 °C for 30 s, followed by 40 cycles of denaturation at 98 °C for 10 s, annealing at 60 °C for 15 s, and extension at 72 °C for 95 s, with a final extension at 72 °C for 5 mins. Cycling was done with a VeritiPro Thermal Cycler (A48141, ThermoFisher Scientific). The resulting PCR products were visualized using a 1% agarose gel containing SYBR™ Safe DNA Gel Stain (S33102, ThermoFisher Scientific) to confirm the presence of a ~3 kb FMDV P1 amplicon based on the TrackIt™ 1 kb Plus DNA Ladder (10488085, ThermoFisher Scientific). PCR products were purified using AMPure XP magnetic beads (A63880, Beckman Coulter, Mississauga, ON, Canada) twice as per the manufacturer’s instructions, with one modification: the ethanol used to wash bound beads was prepared at 80% instead of the recommended 70%. A 0.5× bead-to-PCR product ratio was used for the first bead clean-up step, followed by a 1× ratio for the second bead clean-up step. PCR products were quantified with the Qubit™ 4 fluorometer (Q33226, ThermoFisher Scientific) using the dsDNA BR (Broad Range) Assay Kit (Q32850, ThermoFisher Scientific) as per the manufacturer’s protocol.

### 2.5. Library Preparation, Nanopore Sequencing with Flongle, and Nanopore Data Analysis

For library preparation, approximately 200 ng of purified P1 amplicon was barcoded using the Rapid Barcoding Kit (SQK-RBK114, ONT, Oxford, UK) as per the manufacturer’s instructions with the following modifications: the barcoding incubations at 30 °C and 80 °C were increased from 2 mins to 5 mins, the elution incubation temperature was increased from room temperature to 37 °C, and the adapter ligation incubation time was increased from 5 mins to 20 mins. Once barcoded, amplicons were pooled and purified with AMPure XP beads (1× ratio), resuspended in 6.5 μL of elution buffer, and 1 μL quantified using the Qubit™ 4 fluorometer utilizing the Qubit dsDNA HS (High Sensitivity) Kit (Q32851, ThermoFisher Scientific) as per the manufacturer’s instructions. For sequencing, 0.5 μL of diluted rapid adapter was added to the prepared library and incubated for 20 mins at room temperature.

To produce sequences, the Flongle Flow Cell R10.4.1 (FLO-FLG114, ONT) was used in conjunction with the Flongle adapter (ADP-FLG001, ONT) and MinION Mk1D (ONT) for Nanopore sequencing. Once the flow cell was primed, the entire library was loaded into the flow cell. MinKNOW software was used for data acquisition and basecalling in real-time over 24 h. Basecalling was set to High-Accuracy to filter out reads with quality scores less than 9, and barcode trimming was enabled.

Nanopore sequencing data were processed with Bash as follows: Reads contained in the FASTQ files were mapped using Minimap2 (v2.28) [32] against reference prototype FMDV sequences obtained from The Pirbright Institute [33] with additional SAT1, SAT2, and SAT3 P1 references generated from Illumina sequencing to improve mapping of SAT serotypes (Supplemental Table S1). Mapped reads were sorted, and mapping statistics were computed with Samtools (v1.21) [34]. Samtools was also used to produce a consensus sequence based on the top-matching reference and output information about reference mapping. For consensus sequence generation, minimum read depth at each position of the reference was set to 10×, and a multiplicative correction for heterozygous calling was set to 0.55. The produced information was used to create charts with Plotly Express (v5.22.0) [35]. Consensus sequences were input into the command line version of NCBI BLAST (v2.15.0) [36] for virus identity confirmation and specific FMDV topotype/lineage information. A subset of the BLAST database based on FMDV was used for the NCBI BLAST.

Sequencing “success” was determined using multiple parameters that include the presence of a ~3 kb band visualized by gel electrophoresis after PCR amplification, coverage of the reference genome > 99% at a minimum read depth of 50×, and a minimum of 1000 reads mapped. Another metric based on the inverse proportion of unmapped to total reads was also used to gauge whether a Nanopore sequencing run was successful or not. All samples tested by the FMDV-ONTAPS method were used to determine an appropriate threshold to differentiate positive or negative based on the Receiver Operating Characteristic (ROC) curve analysis through scikit-learn (v1.7.2) [37]. RT-qPCR Cq values were used to classify samples as true positive or true negative.

### 2.6. Sanger Sequencing, Illumina Sequencing, and Data Analysis

Sanger sequencing of the FMDV VP1 gene within the P1 amplicons was conducted as described previously [38]. Briefly, the VP1 region was sequenced using serotype-specific FMDV primers with the BigDye Terminator v3.1 Cycle Sequencing Kit (4337455, ThermoFisher Scientific) and the ABI 3500xL Genetic Analyzer (4404633, ThermoFisher Scientific). Data was analyzed using Geneious Prime version 2023.0.1 (https://www.geneious.com, accessed on 14 May 2023). In short, sequence data from each sample was trimmed with a 5% error probability limit. Forward and reverse sequences were then aligned using MUSCLE alignment version 3.8.425 to generate a consensus sequence. The consensus sequence was then aligned using MUSCLE against the consensus sequence generated from Nanopore sequencing.

Illumina sequencing was performed as previously described [16,39,40]. Briefly, FMDV P1 amplicons were normalized to 0.2 ng/µL with nuclease-free water. Libraries were prepared using the Nextera XT DNA Library Preparation Kit (FC-131-1024, Illumina, San Diego, CA, USA) and Nextera XT Index Kit v2 (FC-131-2001, Illumina) following the manufacturer’s protocol using 20 cycles of PCR during indexing. Libraries were run on the MiSeq System (SY-410-1003, Illumina) using MiSeq Reagent Nano Kit V2 (300-cycles paired end) (MS-102-2002, Illumina) following the manufacturer′s protocol.

Raw Illumina sequencing data were processed with the CFIA-NCFAD/nf-villumina (v.2.0.1) Nextflow workflow [41] for quality control, taxonomic classification, and de novo assembly as done previously [8]. First, nf-villumina removed Illumina PhiX Sequencing Control V3 reads using BBDuk (v38.96) [42], followed by adapter removal and quality filtering with fastp [43]. Filtered reads were retained for de novo assembly with Unicycler (v0.4.8) [44], Shovill (v1.1.0) [45], and MEGAHIT (v1.2.9) [46], and the resulting contigs from each assembly were queried against the online NCBI BLAST nucleotide database (accessed 14 May 2023) [47]. The fastp filtered reads were mapped to the top FMDV BLAST match for each sample in Geneious Prime (v2023.0.1, https://www.geneious.com) (accessed on 14 May 2023) with the Minimap2 (v2.24) [32] assembler on default settings. A 75% majority consensus sequence, approximately 3 kb in length, was called with a low coverage threshold of 10× coverage. The resulting consensus sequence was aligned with the de novo assembled contigs with MAFFT (v7.490) [48] to manually check for assembly errors.

## 3. Results

**Figure 1:**
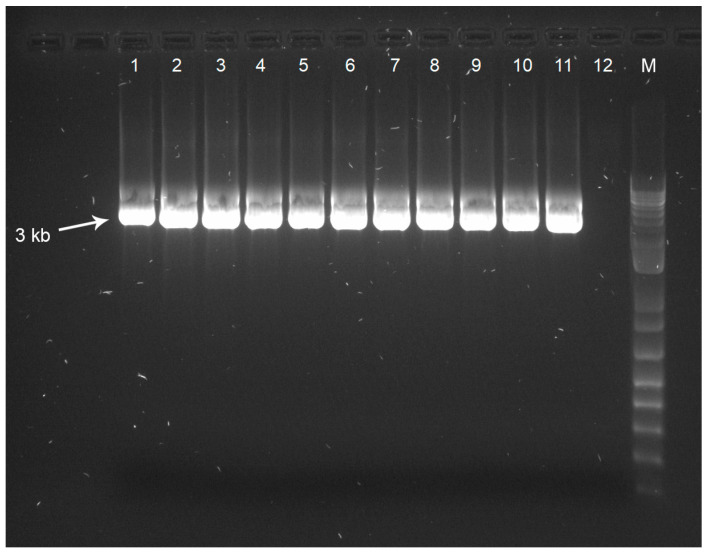
A 1% agarose gel demonstrating the ~3 kb P1 amplicon for representative samples of all seven FMDV serotypes. Samples are as follows: (1) O TUR 1/69, (2) O UKG 1/67, (3) A BRA 1/55, (4) AIRQ 24/64, (5) C SWI 1/65, (6) ASIA1 ISR 1/89, (7) ASIA1 PAK 1/54, (8) SAT1 KEN 4/98, (9) SAT2 ZIM 5/81, (10) SAT2 SAU 1/00, (11) SAT3 ZIM 4/81, and (12) D-PBS as a negative control. (M) indicates the 1 kb+ DNA ladder.

The initial objective of this study was to confirm successful amplification of the P1 region of the FMDV genome and to obtain P1 sequence data from all seven FMDV serotypes. Cell culture isolates representing each of the seven FMDV serotypes were used to assess the effectiveness of the FMDV-ONTAPS protocol. Gel electrophoresis provided visual confirmation of the ~3 kb P1 amplicon, indicating successful amplification of the P1 region from each FMDV serotype (Figure 1).

**Figure 2:**
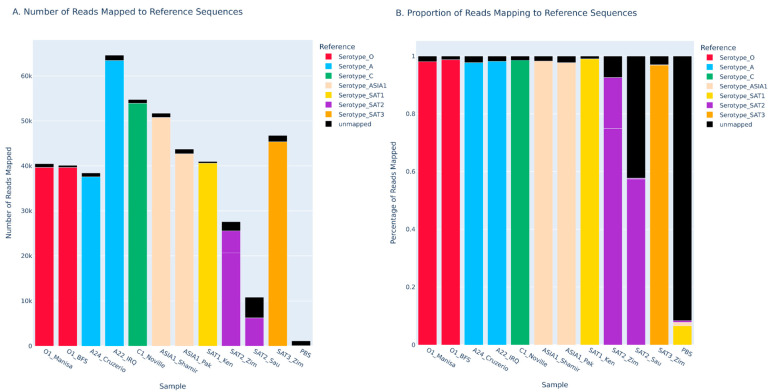
Nanopore sequencing of serotype representative FMDV isolates showing the number of reads mapped to each reference (**A**) and the proportion of the total reads that were mapped to each reference (**B**). Each color corresponds to a different FMDV serotype. Red for O, blue for A, green for C, peach for ASIA1, yellow for SAT1, purple for SAT2, and orange for SAT3.

Corresponding P1 sequence data were also successfully generated from all serotypes with high sequencing quality, such that coverage of the reference sequence at 50× or higher for each sample was >99% (Figure 2, Table 1). This was accomplished in a single sequencing run using the MinION Mk1D platform with the Flongle flow cell and adapter. Each serotype was individually barcoded in a multiplexed library. Samples included O TUR 1/69, O UKG 1/67, A BRA 1/55, A IRQ 24/64, C SWI 1/65, ASIA1 ISR 1/89, ASIA1 PAK 1/54, SAT1 KEN 4/98, SAT2 ZIM 5/81, SAT2 SAU 1/00, and SAT3 ZIM 4/81.

**Table 1:** Mean read depth and coverage of serotype representative FMDV cell culture isolates. The minimum read depth cutoff used is 50or 1% of the mean read depth if this value is greater than 50.

Sample	Reference	Mean Read Depth	Read Depth Cutoff (1% of Mean Read Depth)	% Coverage at Read Depth Cutoff or Greater
O TUR 1/69	AY593823.1	14,932.43	149.32	99.27
O UKG 1/67	AY593815.1	14,129.53	141.30	99.27
A BRA 1/55	AY593768.1	13,695.27	136.95	99.27
A IRQ 24/64	AY593763.1	21,941.99	219.42	99.31
C SWI 1/65	AJ133357.1	20,506.09	205.06	99.27
ASIA1 ISR 1/89	JF739177.1	17,835.41	178.35	99.30
ASIA1 PAK 1/54	AY593795.1	16,673.27	166.73	99.27
SAT1 KEN 4/98	09_SAT1_Ken_consensus	15,345.97	153.46	99.64
SAT2 ZIM 5/81	10_SAT2_Zim_consensus	7959.86	79.60	99.60
SAT2 SAU 1/00	11_SAT2_Sau_consensus	2480.68	50.00	99.64
SAT3 ZIM 4/81	12_SAT3_Zim_consensus	17,665.61	176.66	99.60
D-PBS	09_SAT1_Ken_consensus	26.61	50.00	0.00

Average read depth for each cell culture isolate ranged from 2481 to 21,942 (Table 1). Overall reference coverage for each cell culture isolate was found to be >99% (Table 1).

Consensus sequences produced by the FMDV-ONTAPS protocol were evaluated against established Illumina and Sanger sequencing protocols and found to be comparable. Serotype representative FMDV P1 amplicons were sequenced with Illumina and Sanger sequencing platforms. They produced results showing high percent pairwise identity values (>95%) when compared to consensus sequences produced by FMDV-ONTAPS (Table 2).

To assess the analytical sensitivity of the FMDV-ONTAPS protocol, select FMDV cell culture isolates (one from each serotype) were used to prepare several 10-fold dilution series. These samples were then used to compare the analytical sensitivities of the FMDV-ONTAPS protocol, the FMDV RT-qPCR diagnostic assay, and the DAS ELISA. Total RNA was extracted from each dilution and was tested using RT-qPCR to determine the LOD according to the real-time assay parameters (Cq < 35.99). The LODs observed for the RT-qPCR assay were between 57.89 and 3.72 × 10^3^ RNA copies per 5 μL and varied by serotype (Table 3). For sequencing assessment, P1 amplification was performed on the extracted RNA from each dilution and followed by visualization of the PCR products on an agarose gel prior to Nanopore sequencing (Supplemental Figures S1 and S2). Each sample’s reads mapping to the correct reference was considered to gauge assay performance (Table 3). Coverage data was also generated to support read counts (Supplemental Table S2). Interestingly, the FMDV-ONTAPS protocol was able to correctly generate P1 sequence data when the same sample dilutions were also RT-qPCR positive, demonstrating that positive Cq values (<35.99) are a good indicator of sequencing success. Exceptions to this correlation were observed with four of the seven FMDV serotype representative samples. Specifically, when the SAT2 serotype was evaluated, the RT-qPCR assay was more sensitive and able to detect 3.72 × 10^3^ FMDV genome equivalents. In contrast, the FMDV-ONTAPS protocol’s LOD was 10-fold lower at 5.78 × 10^4^ FMDV genome equivalents (Table 3F). On the contrary, the FMDV-ONTAPS protocol was more sensitive than the RT-qPCR assay when serotypes O, A, and SAT1 were tested (Table 3A,B,E). The FMDV-ONTAPS protocol was able to detect samples that had 10-fold more FMDV genome equivalents in comparison to the RT-qPCR for those serotypes. Nanopore sequencing of all other representative serotype samples showed sensitivity equivalent to or better than the RT-qPCR (Table 3). All samples in the serial dilution series were also tested by the DAS ELISA to evaluate the sensitivity in comparison to the other tests (Table 3). As predicted, the DAS ELISA was far less sensitive than the FMDV-ONTAPS protocol and RT-qPCR assay. LOD data produced by the DAS ELISA were between 10^3^ and 10^5^-fold lower than those produced by the two molecular-based assays (Table 3). Reference coverage also supports the sensitivity limits of the FMDV-ONTAPS protocol. Each sample that had a meaningful number of reads mapped had >99% coverage of the reference (Supplemental Tables S3 and S4). ROC curve analysis of all samples sequenced using the FMDV-ONTAPS protocol produced a curve with an area under the curve of 0.9602–indicating a well-fitting model (Supplemental Figure S3). The suggested threshold based on the curve is 0.6465 (Supplemental Table S5); however, the value of 0.30 was chosen due to the presence of known positive samples with an inverse proportion of unmapped reads to total reads between 0.30 and 0.6465. All negative samples fell far below 0.30 (Supplemental Table S6).

**Figure 3:**
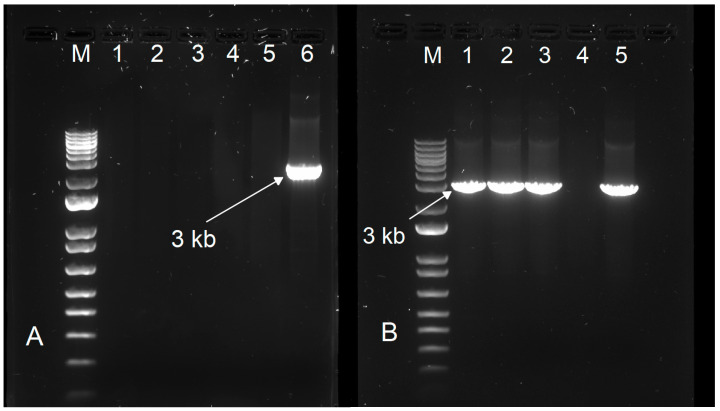
Gel electrophoresis analysis of differential vesicular disease virus samples processed with the FMDV-ONTAPS protocol. (**A**) Electrophoresis of individual non-target vesicular disease viruses: (1) SVDV PRT 1/03, (2) VSIV 85CLB, (3) VSNJV 92CLB, (4) SVA CAN 1/15, (5) D-PBS, and (6) FMDV A IRQ 24/64. (**B**) Electrophoresis of a 1:1 extracted RNA mixture for FMDV with differential vesicular disease viruses. (1) FMDV A IRQ 24/64 mixed with SVDV PRT 1/03, (2) FMDV A IRQ 24/64 mixed with VSIV 85CLB, (3) FMDV A IRQ 24/64 mixed with SVA CAN 1/15, (4) D-PBS, and (5) FMDV A IRQ 24/64 only. (M) indicates the 1 kb+ DNA ladder.

**Figure 4:**
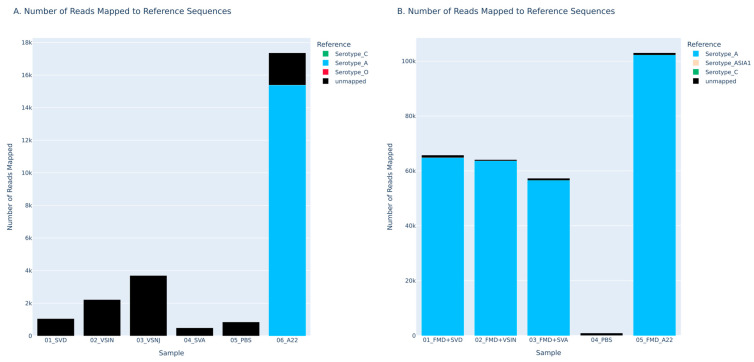
Nanopore sequencing analysis of differential vesicular disease viruses and mixed infections processed with the FMDV-ONTAPS protocol. Read-mapping results for (**A**) individual non-target differential vesicular disease viruses and (**B**) 1:1 extracted RNA mixtures of FMDV with differential disease viruses. The blue indicates reads that mapped to FMDV serotype A reference sequences. Black represents the number of reads that did not map against any FMDV reference sequence.

To evaluate the analytical specificity, the FMDV-ONTAPS protocol was assessed against a panel of differential vesicular disease viruses that included Swine Vesicular Disease Virus (SVDV), Vesicular Stomatitis Indiana Virus (VSIV), Vesicular Stomatitis New Jersey Virus (VSNJV), and SenecaVirus A (SVA). Gel electrophoresis confirmed the absence of amplicon for all non-target, differential viruses (Figure 3A), and subsequent nanopore sequencing detected only unmapped reads present in all these samples, with the exception of the FMDV positive control (Figure 4A). To further assess analytical specificity in mixed infections, 1:1 RNA mixtures of differential vesicular disease viruses with FMDV were tested with the FMDV-ONTAPS protocol. Gel electrophoresis confirmed the presence of the expected ~3 kb FMDV-specific amplicon in all mixed samples containing FMDV RNA (Figure 3B), and nanopore sequencing showed that the majority of reads mapped to FMDV reference sequences (Figure 4B).

**Figure 5:**
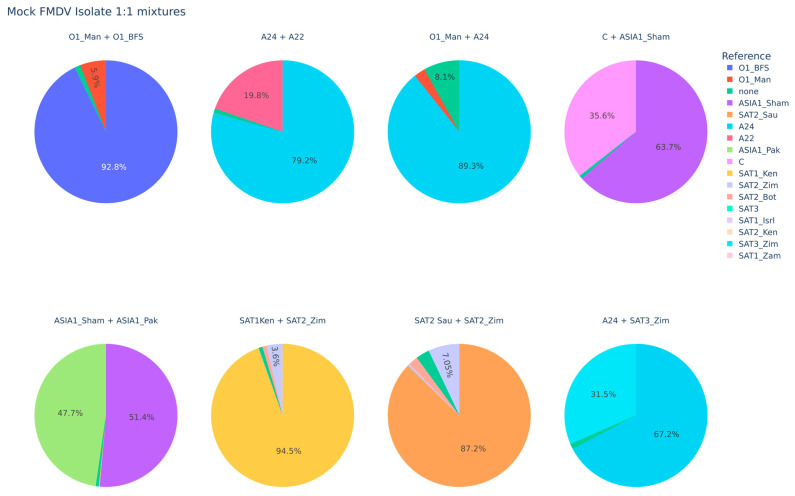
FMDV-ONTAPS of various FMDV serotype/strain combinations, showing the percentage of reads mapping to a particular reference. Values less than 5% are not shown.

The FMDV-ONTAPS protocol was also tested for the ability to resolve co-infections of different FMDV serotypes or strains. The results are variable–some combinations produced disproportionate representation of one serotype or strain, while other combinations resulted in more balanced representation (Figure 5).

Next, the ability of the FMDV-ONTAPS protocol’s performance was evaluated using a variety of FMDV-containing sample types. Multiple sample matrices were assessed including milk (n = 4), serum (n = 4), oral swabs (n = 6), nasal swabs (n = 6), tissue suspensions (n = 6), vesicular fluid (n = 2), and oral fluid (n = 6) obtained as remnant samples from a variety of animal experiments. All samples were tested with the RT-qPCR assay to obtain Cq values prior to P1 amplification. Sample Cqs ranged from very low (Cq = 8 for vesicular fluid) up to 28.12 (Table 4).

**Figure 6:**
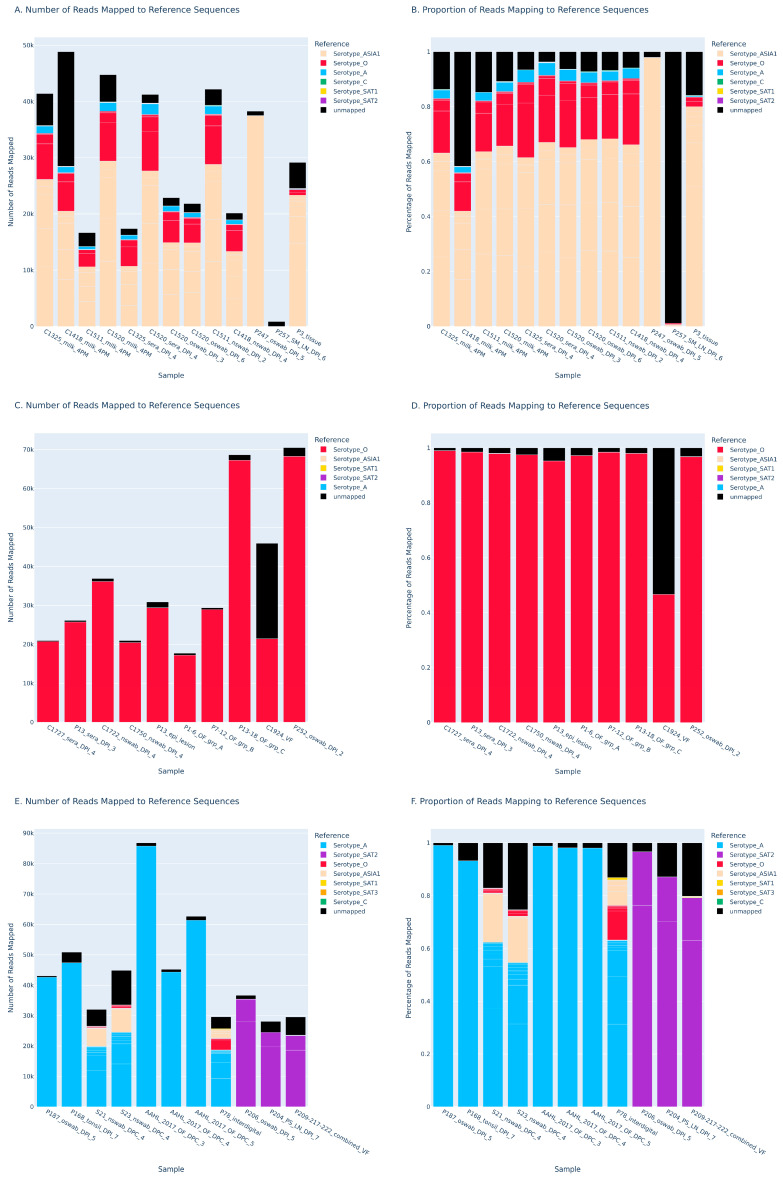
Nanopore sequencing of various sample matrices from animals experimentally infected with FMDV ASIA1 (**A**,**B**), O (**C**,**D**), and A or SAT2 serotypes (**E**,**F**). Sequencing shows the number of reads output that mapped to reference sequences (**A**,**C**,**E**) and the overall proportion of reads that mapped to each reference (**B**,**D**,**F**). The peach color depicts reads that are mapped to ASIA1 reference sequences, red depicts reads that are mapped to O, blue is for reads that are mapped to A, and purple is for reads that are mapped to SAT2.

Thirty-three of the thirty-four various samples tested satisfied positivity metrics based on reads mapped greater than 1000, reference coverage greater than 99% (Supplemental Table S2), and an inverse unmapped to total read proportion greater than 30% (Supplemental Table S6) with FMDV-ONTAPS protocol apart from a single sample (p257 submandibular lymph node tissue suspension, FMD-ASIA1 ISR 1/89) with a RT-qPCR Cq = 28.21 (Figure 6, Table 4). Tissue types range from milk, serum, oral swabs, nasal swabs, oral fluids, vesicular fluid, and various tissues.

The experimental sample with the highest Cq value to generate the P1 amplicon and sequence was a nasal swab with a Cq = 27.26 (FMDV-A). Samples collected from cattle experimentally infected with ASIA1 PAK 9/14 and sequenced had an average of 63.07% of reads mapping to the correct serotype, while cattle experimentally infected with O UKG 11/01 averaged 85.26% of reads mapping to the correct serotype. Nasal swabs from sheep experimentally infected with A VIT 15/12 averaged 58.49%. Interdigital tissue from pig 78 (P78) also showed a lower percentage (63.25%) of reads mapping to the correct serotype compared to other serotypes of FMDV and other tissue types in the same animal model. Among the remaining sample types and animal models tested, the percentage of reads mapping to the correct serotype averaged much higher at 92.65%. Read depth and coverage data further support the sequencing results with >99% coverage of the reference sequence and high mean read depths (Table S2). The singular sample (p257 submandibular lymph node) that failed to generate sequencing data using the FMDV-ONTAPS protocol did not produce a visible amplicon (Figure S2).

**Figure 7:**
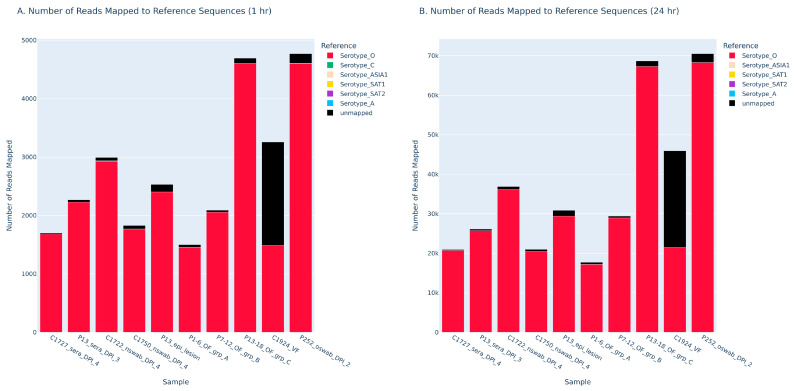
Comparison of the FMDV-ONTAPS protocol with data at 1 h (**A**) and 24 h (**B**) post sequencing start, showing the number of reads mapped. Sample matrices assessed include serum, nasal swabs, epithelial lesions, oral fluids, vesicular fluid, and oral swabs. These samples are from animals experimentally infected with FMDV O. Reads that mapped to serotype O references are shown in red.

The FMDV-ONTAPS protocol also yielded information on FMDV serotype identification within 1 hour (h) of sequencing start (Figure 7). All samples assessed this way were from previous animal experiments involving FMDV O UKG 11/01 or O TUR 1/69. All samples were correctly identified as FMDV serotype O both at 1 h post sequencing start, and at 24 h post sequencing start. Samples passed quality metrics for the presence of ~3 kb fragment on an agarose gel (Figure S2), read depth, reference coverage, and inverse proportion of unmapped to total reads (Table 5 and Table 6). Notably, the ratio of mapped reads to total reads for each sample is relatively the same (Table 5). Only a marginal difference of 2.46% (0.0246) in the proportion of mapped reads to total reads is observed for the vesicular fluid sample (C1924_VF_4dpi).

## 4. Discussion

Nanopore sequencing is a multi-step process that includes nucleic acid extraction of samples, library preparation, sequencing, and bioinformatic analysis. Nanopore sequencing allowed rapid and accurate identification of the FMDV serotype, topotype, and lineage. To determine if the FMDV-ONTAPS protocol produced results comparable to established sequencing protocols, serotype representative FMDV P1 amplicons were sequenced using Illumina and Sanger sequencing platforms.

The FMDV-ONTAPS protocol was found to have quicker turnaround times and is less expensive than the other sequencing methods. The FMDV-ONTAPS protocol has the advantage over Sanger sequencing because it does not require knowledge of the FMDV serotype. This significantly simplifies the sequencing process and results in overall turnaround times as short as 6 to 7 h with the FMDV-ONTAPS procedure after initial RNA extraction. In contrast, Sanger sequencing would require a minimum of 12 h from extracted RNA to sequence data in the best-case scenario. Turnaround times can be delayed up to 3 days if the serotype is not known. FMDV-ONTAPS performance was found to be equivalent to Sanger and Illumina sequencing while significantly surpassing the limitations of the DAS ELISA in terms of sensitivity (Table 2 and Table 3). Illumina sequencing has the edge over Nanopore sequencing due to higher read quality scores. However, the library preparation process with Illumina sequencing is lengthier. The reagents needed, and initial capital investment costs, are also greater for Illumina sequencing than with Nanopore sequencing–especially if Flongle flow cells are used. The advantage of Nanopore sequencing is greater read depth at a fraction of the cost and the faster turnaround time to obtain results [49]. Greater read depth with Nanopore sequencing was observed compared to Illumina sequencing for the representative FMDV cell culture isolates, except for SAT2 ZIM 5/81 and SAT2 SAU 1/00 (Supplemental Table S4). This could be due to the use of a nano flow cell for the Illumina sequencing. Data output from the Illumina nano flow cell can be as high as 500 Mb [50], whereas the ONT Flongle flow cell can produce data up to 2.8 Gb [51].

A major disadvantage of the Flongle flow cell is the short shelf life. ONT warranty covers up to 4 weeks upon receipt; any Flongle flow cells used past this time frame typically did not perform well due to pore loss, ONT has also made the decision to discontinue the Flongle flow cell and the associated adapter for new customers. This product is now only available to legacy users. The MinION flow cells remain available as a more expensive alternative that is known to have better performance due to its higher throughput and longer shelf life over Flongle flow cells.

The biggest criticism of ONT-based sequencing in comparison to other technologies has been higher error rates of up to 10% with earlier iterations of the flow cell and library preparation chemistry [52]. However, newer versions of the flow cell (R10) use improved chemistry and pore technology that have resulted in reports of base calling accuracies up to 99% and an improvement in average Phred scores [52,53]. The updates made to the R10 flow cells have also improved sequencing accuracy of homopolymer regions [53]. Regular updates to ONT sequencing improve performance, but make validation of diagnostic assays challenging. However, any future changes to the sequencing technology can be evaluated quickly, provided the changes are not drastic.

The analytical sensitivity data demonstrate that the FMDV-ONTAPS protocol is highly sensitive (Table 3). Based on the data presented in this study, if a diagnostic sample tested FMDV positive by the RT-qPCR assay, the FMDV-ONTAPS protocol would reliably produce the P1 amplicon and sequence data, allowing for serotype identification irrespective of the sample type.

The analytical specificity of the FMDV-ONTAPS protocol demonstrates that the assay reliably detects FMDV without cross-reacting with other non-target vesicular disease viruses (Figure 3 and Figure 4). In contrast, the performance of the protocol in simulated FMDV-specific serotype/strain co-infection scenarios was variable (Figure 5). While some FMDV serotype/strain combinations resulted in relatively balanced representation in the sequencing analysis, others produced data where one serotype/strain was overrepresented, despite mixing the RNA at a 1:1 ratio (Figure 5).

The threshold for inverse proportion of unmapped to total reads was determined using values from all samples assessed by the FMDV-ONTAPS protocol. This metric was chosen to account for sequencing runs that produced a high number of unmapped reads or a low number of total reads. For a threshold of this metric, the ROC curve cutoff suggestion of 0.6465 was not used and instead lowered to 0.30 due to known positive samples falling in the 0.30 to 0.6465 range (Supplemental Figure S3, Supplemental Tables S5 and S6). The ROC curve generated could be more accurate with further assessment of negative samples, as this was limited in this study (n = 18).

Analysis of sample matrices demonstrated that all sample types tested are suitable for amplification and sequencing of the FMDV P1 gene, with the caveat that viral load impacts assay sensitivity compared to the ideal conditions of serially diluted cell-cultured virus. From the subset of matrices assessed, it appears that host species, FMDV serotype, and the sample matrix affect the performance of the FMDV-ONTAPS protocol. Samples from cattle experimentally infected with O UKG 11/01 had a higher proportion of reads mapping to the correct serotype compared to cattle experimentally infected with ASIA1 PAK 9/14 (Table 4). Samples where pigs are the host species, in general, saw a higher proportion of reads mapping to the correct serotype (Table 4). This infers the possibility that host species, virus serotype, and sample matrices beyond those tested could impact the success of the FMDV-ONTAPS protocol. More comprehensive studies with statistically significant representation from each animal model, FMDV serotype, and sample matrix would be needed for confirmation.

Table 3 demonstrates that the FMDV-ONTAPS protocol’s LOD for most serotypes of FMDV is equivalent to the RT-qPCR LOD except for the SAT2 ZIM 5/81 isolate. An overall lower sensitivity was observed with the FMDV-ONTAPS protocol in comparison to the RT-qPCR, specifically for the SAT2 ZIM 5/81 isolate. Agarose gel images show visible bands up to their respective 10-fold dilutions that correspond to the LOD seen with Nanopore sequencing (Supplemental Figure S1). Therefore, the sensitivity limitation is at the PCR amplification step and not the Nanopore sequencing workflow itself.

Based on preliminary findings, the RT-qPCR-based LOD cut-off for success with the FMDV-ONTAPS protocol for all sample matrices tested was 28. However, a definitive Cq threshold cannot be established for all sample types without further investigation. Samples with a Cq value of 25 or less resulted in successful P1 amplification, whereas samples with Cq values greater than 25 demonstrated variable P1 amplification. Genome coverage and the inverse proportion of unmapped reads to total reads of matrices samples with successful P1 amplification also tells the same story: Coverage at 50× was >99% for all samples except for the one sample that did not amplify (0% coverage), and the average inverse ratio of unmapped to total reads for positive samples is 0.9045 (Supplemental Tables S2 and S5). The inverse ratio of unmapped to total reads for the single sample (p257 Submandibular Lymph Node 6 dpi) that was not successful with P1 amplification is 0.0116 and falls below the ~0.30 threshold (Supplemental Table S5). Samples with Cq values greater than 30 were not tested.

Complexities were encountered while assessing the FMDV-ONTAPS protocol. From a wet-laboratory perspective, difficulties included reduced sensitivity for certain samples, sample age limitations, and potential bias in detecting specific serotypes/strains in simulated FMDV co-infection scenarios. Reduced sensitivity could be attributed to long-term storage of samples. Extracted RNA from cell culture isolates stored for >6 months exhibited reduced amplification by PCR and thus reduced sensitivity by the FMDV-ONTAPS protocol. The most likely explanation is RNA degradation even with storage at −70 °C. This can also explain the reduced sensitivity observed with the different sample types tested, as these specimens were stored for long periods (>1 year) prior to use. However, these variables would not affect specimens submitted for diagnostic testing, as these samples would be processed immediately upon receipt. For mock co-infection experiments involving multiple FMDV serotypes or strains, resolution of specific serotype/strain proved difficult in some instances, as there may be bias introduced by the primers in amplicons generated from certain FMDV serotypes/strains (Figure 5).

From the data analysis perspective, a high-performance computer, command line expertise with bash and Python, and the installation of all open-source software were challenging to troubleshoot. Live basecalling of reads generated through Nanopore sequencing required a computer with a graphics processing unit having a minimum of 8 Gigabytes (GB) of memory. Graphics processing units with less than 8 GB cannot basecall reads as they are produced. Therefore, this requirement is essential for quick turnaround times. As all data produced from Nanopore sequencing is analyzed with the command line, knowledge of coding with bash is essential. Administrative privileges are required for utilizing and installing all necessary programs to analyze sequencing results. Computers without these privileges, such as those with organization-specific restrictions, result in complications.

While there is reduced sensitivity for the detection of FMDV observed in different sample matrices, Nanopore sequencing is still a viable option with samples that do not produce meaningful results by the DAS-ELISA, given the observed differences in test sensitivity with FMDV isolates (Table 3). The FMDV-ONTAPS protocol also demonstrated strong analytical specificity to FMDV. No amplification was observed for any of the non-target differential vesicular disease viruses (Figure 3A), and no nanopore reads from the differentials viruses mapped to any FMDV reference sequence (Figure 4A), including mock mixture experiments (Figure 4B). The FMDV-ONTAPS protocol is a powerful tool not only to accurately provide the serotype and topotype/lineage of the FMDV present, but also to aid in emergency vaccine selection to combat outbreaks. Furthermore, providing a greater number of P1 sequences allows for the generation of more information about the vast genetic diversity and evolution of this highly contagious virus. This is particularly important to monitor disease spread.

This study demonstrates the feasibility and accuracy of using the FMDV-ONTAPS protocol for rapid sequencing of FMDV, providing a valuable and affordable protocol that can support diagnostic testing and genomics research. In the event of a FMDV incursion, rapid sequencing using the MinION Mk1D platform offers a highly effective diagnostic solution as it can provide accurate FMDV serotype identification and data to support outbreak tracing and informed vaccine selection. The FMDV-ONTAPS protocol utilizing the ONT MinION Mk1D can easily be implemented into diagnostic workflows to allow for rapid and accurate identification of FMDV serotype. Furthermore, the analytical sensitivity data provide evidence that the sensitivity of the FMDV-ONTAPS protocol is much greater than that of the conventional DAS ELISAs (Table 3). MinKNOW software supports real-time basecalling, enabling results to be generated within one hour of the sequencing initiation (Figure 7, Table 5) and thereby reducing turnaround times in urgent situations. Therefore, this study supports the replacement of the DAS ELISA with the FMDV-ONTAPS protocol for FMDV serotype identification.

## Figures and Tables

**Table 2:** Pairwise comparisons between Illumina and Nanopore sequencing platforms, and Sanger and Nanopore sequencing platforms, using serotype representative FMDV cell culture isolates. Alignments between each consensus sequence were produced using MUSCLE alignment to determine pairwise identity.

Sample	% Pairwise Identity (Illumina vs. Nanopore)	% Pairwise Identity (Sanger vs. Nanopore)
**O TUR 1/69**	100%	99.3%
**O UKG 1/67**	100%	99.5%
**A BRA 1/55**	99.9%	99.4%
**A IRQ 24/64**	100%	99.9%
**C SWI 1/65**	100%	100%
**ASIA1 ISR 1/89**	100%	99.9%
**ASIA1 PAK 1/54**	99.9%	99.9%
**SAT1 KEN 4/98**	95.8%	95.7%
**SAT2 ZIM 5/81**	100%	99.9%
**SAT2 SAU 1/00**	99.9%	99.6%
**SAT3 ZIM 4/81**	99.5%	99.3%

**Table 3:** Analytical sensitivity of the FMDV RT-qPCR (highlighted in orange, Cq threshold of 35.99), DAS ELISA (highlighted in turquoise, OD threshold of 0.1), and Nanopore sequencing (highlighted in blue, showing the number of reads mapped to the reference sequence of the correct serotype) using 10-fold dilutions of serotype representative FMDV cell culture isolates. Approximate copy numbers were generated based on a standard curve of a known quantity and the copy number of in vitro transcribed FMDV 3D gene RNA per 5 uL. “*” indicates an estimation of the copy number based on 10-fold dilutions. Extrapolated copy numbers less than 1 are shown as “NA”.

**(A) O UKG 1/67**			
**Approximate RNA Copy Number**	**FMDV RT-qPCR Cq**	**DAS ELISA OD**	**Number of Reads Mapped**
3.09 × 10^7^	14.44	1.37	39,017
3.32 × 10^6^	17.77	0.19	56,379
3.84 × 10^5^	20.99	0.01	26,311
3.29 × 10^4^	24.66	0.00	50,482
3.51 × 10^3^	28.00	0.00	17,882
268.01	31.84	0.00	15,008
6.73	37.34	0.00	8362
NA	Undetermined	0.00	6
**(B) A BRA 1/55**			
**Approximate RNA Copy Number**	**FMDV RT-qPCR Cq**	**DAS ELISA OD**	**Number of Reads Mapped**
9.39 × 10^7^	12.78	1.47	42,609
7.93 × 10^6^	16.47	0.27	12,430
7.40 × 10^5^	20.01	0.00	33,085
6.25 × 10^4^	23.70	0.00	10,265
7.79 × 10^3^	26.81	0.00	23,115
579.01	30.69	0.00	19,601
3.31	38.40	0.00	8196
NA	Undetermined	0.00	34
**(C) C SWI 1/65**			
**Approximate RNA Copy Number**	**FMDV RT-qPCR Cq**	**DAS ELISA OD**	**Number of Reads Mapped**
2.12 × 10^7^	15.00	2.27	88,444
1.80 × 10^6^	18.68	0.52	134,322
1.71 × 10^5^	22.20	0.06	65,470
1.48 × 10^4^	25.85	0.01	108,037
1.28 × 10^3^	29.51	0.02	44,122
9.03	36.90	0.01	53
NA	Undetermined	0.01	54
NA	Undetermined	0.00	49
**(D) ASIA1 PAK 16/14**			
**Approximate RNA Copy Number**	**FMDV RT-qPCR Cq**	**DAS ELISA OD**	**Number of Reads Mapped**
1.85 × 10^7^	13.58	0.81	77,454
2.18 × 10^6^	17.10	0.19	43,094
1.95 × 10^5^	20.57	0.04	61,547
2.08 × 10^4^	24.14	0.01	36,276
2.24 × 10^3^	27.69	0.00	48,013
57.89	34.44	0.00	20,830
5.79 *	Undetermined	0.00	7
NA	Undetermined	0.00	15
**(E) SAT1 KEN 4/98**			
**Approximate RNA Copy Number**	**FMDV RT-qPCR Cq**	**DAS ELISA OD**	**Number of Reads Mapped**
4.67 × 10^7^	13.82	0.67	55,643
3.98 × 10^6^	17.50	0.10	66,032
3.35 × 10^5^	21.19	0.04	62,519
3.85 × 10^4^	24.43	0.03	47,272
2.71 × 10^3^	28.39	0.00	40,102
124.96	36.42	0.00	16,512
12.50 *	Undetermined	0.00	13
1.25 *	Undetermined	0.00	11
**(F) SAT2 ZIM 5/81**			
**Approximate RNA Copy Number**	**FMDV RT-qPCR Cq**	**DAS ELISA OD**	**Number of Reads Mapped**
6.04 × 10^7^	13.44	0.31	64,254
5.43 × 10^6^	17.04	0.06	41,331
5.35 × 10^5^	20.50	0.02	50,806
5.78 × 10^4^	23.82	0.03	6550
3.72 × 10^3^	27.91	0.03	5
15.30	36.12	0.03	4
1.53 *	Undetermined	0.03	11
NA	Undetermined	0.03	46
**(G) SAT3 ZIM 4/81**			
**Approximate RNA Copy Number**	**FMDV RT-qPCR Cq**	**DAS ELISA OD**	**Number of Reads Mapped**
3.53 × 10^7^	14.24	1.91	52,168
3.17 × 10^6^	17.84	0.45	72,751
2.71 × 10^5^	21.51	0.05	47,394
3.24 × 10^4^	24.68	0.03	60,932
3.75 × 10^3^	27.90	0.02	32,250
217.46	32.15	0.03	27,595
21.75 *	Undetermined	0.00	25
2.18 *	Undetermined	0.02	17

**Table 4:** Nanopore sequencing and RT-qPCR results of various sample types from animals experimentally infected with FMDV. Nanopore sequencing is depicted based on the percentage of reads that mapped to the correct FMDV serotype, while RT-qPCR is expressed in terms of Cq.

Sample	Sample Type	FMDV Isolate	Cq	% Reads Mapped to Correct Serotype
C1325 milk 4 dpi	milk	ASIA1 PAK 9/14	16.20	63.12%
C1418 milk 4 dpi	milk	ASIA1 PAK 9/14	13.78	41.99%
C1511 milk 4 dpi	milk	ASIA1 PAK 9/14	22.46	63.66%
C1520 milk 4 dpi	milk	ASIA1 PAK 9/14	19.28	65.69%
C1325 serum 4 dpi	serum	ASIA1 PAK 9/14	20.45	61.49%
C1520 serum 4 dpi	serum	ASIA1 PAK 9/14	14.66	67.04%
C1520 o.swab 3 dpi	oral swab	ASIA1 PAK 9/14	18.40	65.14%
C1520 o.swab 6 dpi	oral swab	ASIA1 PAK 9/14	19.02	68.08%
C1511 n.swab 2 dpi	nasal swab	ASIA1 PAK 9/14	15.42	68.34%
C1418 n.swab 4 dpi	nasal swab	ASIA1 PAK 9/14	19.57	66.18%
P247 O.Swab 5 dpi	oral swab	ASIA1 ISR 1/89	26.85	97.95%
P257 Submand.LN 6 dpi	tissue (SM LN)	ASIA1 ISR 1/89	28.21	0.01%
P3 tissue	epithelial	ASIA1 PAK 20/03	11.63	80.04%
C1727 serum 4 dpi	serum	O UKG 11/01	17.77	98.97%
P13 serum 3 dpi	serum	O UKG 11/01	24.27	98.49%
C1722 n.swab 4 dpi	nasal swab	O UKG 11/01	21.53	97.92%
C1750 n.swab 4 dpi	nasal swab	O UKG 11/01	20.50	97.49%
P13 epi.lesion	tissue (ves lesion)	O UKG 11/01	13.38	95.22%
P1–6 GRP A OF	oral fluids	O UKG 11/01	19.96	97.17%
P7–12 GRP B OF	oral fluids	O UKG 11/01	21.09	98.43%
P13–18 GRP C OF	oral fluids	O UKG 11/01	19.84	98.00%
C1924 ves.fluid	vesicular fluid	O UKG 11/01	7.35	46.65%
P252 O.Swab 2 dpi	oral swab	O TUR 1/69	20.30	96.80%
P187 O.Swab 2 dpi	oral swab	A IRQ 24/64	16.49	99.01%
P168 Tonsil 7 dpi	tissue (tonsil)	A IRQ 24/64	23.21	93.21%
S21 n.swab 4 dpi	nasal swab	A VIT 15/12	27.26	62.33%
S23 n.swab 4 dpi	nasal swab	A VIT 15/12	24.82	54.65%
P GRP OF 3 dpi	oral fluids	A IRQ 24/64	16.23	98.76%
P GRP OF 4 dpi	oral fluids	A IRQ 24/64	19.93	98.06%
P GRP OF 5 dpi	oral fluids	A IRQ 24/64	22.41	97.97%
P78 Interdigital	tissue (interdigital)	A IRN 1/09	22.50	63.25%
P206 O.Swab 5 dpi	oral swab	SAT2 ZIM 5/81	17.87	96.52%
P204 Prescap.LN 7 dpi	tissue (prescap LN)	SAT2 ZIM 5/81	24.37	87.12%
P209 P217 P222 combined VF	vesicular fluid	SAT2 ZIM 5/81	7.26	79.26%

Note: Animal species for each sample is indicated by “C” for cattle, “P” for pigs, and “S” for sheep. SM LN = submandibular lymph node, ves lesion = vesicular lesion, and prescap LN = prescapular lymph node.

**Table 5:** Proportion of mapped reads to total reads at 1 and 24 h post sequencing start, and the absolute value of the difference between the two time points for FMDV O samples from experimentally infected animals.

Sample	1 h	24 h	Difference
C1727 serum 4 dpi	0.9838	0.9827	0.0011
P13 serum 3 dpi	0.9842	0.9850	0.0009
C1722 n.swab 4 dpi	0.9231	0.9174	0.0058
C1750 n.swab 4 dpi	0.9758	0.9773	0.0015
P13 epi.lesion 3 dpi	0.9079	0.9072	0.0007
P1–6 GRP A OF 3 dpi	0.9870	0.9879	0.0008
P7–12 GRP B OF 3 dpi	0.9540	0.9501	0.0039
P13–18 GRP C OF 3 dpi	0.9825	0.9784	0.0041
C1924 ves.fluid 4 dpi	0.3617	0.3371	0.0246
P1–6 GRP A OF 3 dpi	0.9702	0.9669	0.0033
D-PBS	0.0460	0.0486	0.0026

Note: Animal species for each sample is indicated by “C” for cattle and “P” for pigs.

**Table 6:** Quality metrics for representative FMDV O samples from experimentally infected animals at 1 h (yellow) and 24 h (green) post sequencing start. Metrics are read depth, reference coverage at minimum read depth (50× or 1% of the mean read depth, whichever is higher), and inverse proportion of unmapped to total reads.

**Sample (1 h)**	**Mean Read Depth**	**Min. Read Depth**	**Coverage**	**Inverse Unmapped to Total Read Ratio**
C1727 serum 4 dpi	1935.02	50.00	99.24	0.9836
P13 serum 3 dpi	2795.58	50.00	99.24	0.9842
C1722 n.swab 4 dpi	1288.07	50.00	99.24	0.9231
C1750 n.swab 4 dpi	1959.40	50.00	99.24	0.9758
P13 epi.lesion 3 dpi	1021.85	50.00	99.24	0.9047
P1–6 OF GRP A 3 dpi	1683.40	50.00	99.24	0.9870
P7–12 OF GRP B 3 dpi	1337.87	50.00	99.24	0.9525
P13–18 OF GRP C 3 dpi	927.16	50.00	99.24	0.9814
C1924 ves.fluid 4 dpi	651.23	50.00	99.24	0.3556
P252 o.swab dpi 2	927.04	50.00	99.24	0.9702
D-PBS	0.9782	50.00	0.00	0.0460
**Sample (24 h)**	**Mean Read Depth**	**Min. Read Depth**	**Coverage**	**Inverse Unmapped to Total Read Ratio**
C1727 serum 4 dpi	25,988.01	259.88	99.27	0.9827
P13 serum 3 dpi	38,713.99	387.14	99.27	0.9850
C1722 n.swab 4 dpi	16,001.16	160.01	99.40	0.9174
C1750 n.swab 4 dpi	28,029.02	280.29	99.27	0.9773
P13 epi.lesion 3 dpi	12,912.59	129.13	99.27	0.9072
P1–6 OF GRP A 3 dpi	20,790.84	207.91	99.34	0.9879
P7–12 OF GRP B 3 dpi	16,443.03	164.43	99.27	0.9501
P13–18 OF GRP C 3 dpi	11,192.16	111.92	99.37	0.9784
C1924 ves.fluid 4 dpi	7013.01	70.13	99.64	0.3371
P252 o.swab dpi 2	23,427.82	234.28	99.34	0.9669
D-PBS	1.32	50.00	0.00	0.0486

Note: Animal species for each sample is indicated by “C” for cattle and “P” for pigs.

## Data Availability

The original contributions presented in this study are included in the article/Supplementary Materials. Further inquiries can be directed to the corresponding author(s).

## Supplementary Materials

The following supporting information can be downloaded at: https://www.mdpi.com/article/10.3390/v18040418/s1, Table S1: FMDV P1 consensus sequences produced from Illumina sequencing and used as references for mapping of Nanopore sequencing reads; Figure S1: A 1% agarose gel demonstrating P1 amplification of 10-fold diluted representative FMDV cell culture isolates; Figure S2: A 1% agarose gel demonstrating P1 amplification of FMDV matrices samples from experimentally infected animals; Table S2: Mean read depth and coverage of various 10-fold diluted FMDV cell culture isolates. Minimum read depth cutoff used is 50× or 1% of the mean read depth if the value is greater than 50; Table S3: Mean read depth and coverage of various clinical FMDV samples from experimentally infected animals. Minimum read depth cutoff used is 50× or 1% of the mean read depth if the value is greater than 50; Table S4: Mean read depth of various FMDV isolates sequenced via Nanopore (flongle flow cell) and Illumina (nano flow cell) sequencing; Figure S3: Receiver Operating Characteristic (ROC) curve using the RT-qPCR as the determiner for positive or negative samples to measure false positive rate and true positive rate of the inverse ratio of unmapped reads to total reads generated by the FMDV-ONTAPS protocol. Area Under the Curve (AUC) value is 0.96 and indicates a good fitting model; Table S5: FMDV-ONTAPS protocol thresholds for each False Positive Rate (FPR) and True Positive Rate (TPR); Table S6: PCR positive (1) or negative (0), number of unmapped reads, total number of reads, and the inverse proportion of unmapped to total reads for each sample evaluated with the FMDV-ONTAPS protocol; Table S7: Cost per sample, turnaround time, sensitivity, and equipment cost comparisons between Sanger, Illumina, and Nanopore Sequencing methods; Supplemental protocol: Bash analysis commands used to produce results for the FMDV-ONTAPS protocol.

## Abbreviations

The following abbreviations are used in this manuscript:

FMD	Foot-and-Mouth Disease
FMDV	Foot-and-Mouth Disease Virus
RT-qPCR	Reverse Transcription quantitative Polymerase Chain Reaction
DAS-ELISA	Double Antibody Sandwich Enzyme-Linked Immunosorbent Assay
SAT	South African Territories
SVDV	Swine Vesicular Disease Virus
SVA	Senecavirus A
VSV	Vesicular Stomatitis Virus
ONT	Oxford Nanopore Technologies
NGS	Next Generation Sequencing
FMDV-ONTAPS	FMDV Oxford Nanopore Technologies Amplicon P1 Sequencing
ROC	Receiver Operating Characteristic
LOD	Limit of Detection
D-PBS	Dulbecco’s Phosphate Buffered Saline
