# Supplementary material for: Rapid Sequence Identification of Foot-and-Mouth Disease Virus Utilizing FMDV-ONTAPS: The Oxford Nanopore Technologies Amplicon P1 Sequencing Protocol
Source: Viruses. 2026 Mar 28;18(4):418. doi: 10.3390/v18040418 (PMC13120243; doi:10.3390/v18040418)
Supplement: Supplementary file 1 [file viruses-18-00418-s001.zip › viruses-4127959-supplementary.pdf]

Supplementary Materials

Table S1. FMDV P1 consensus sequences produced from Illumina sequencing and used as references for mapping of Nanopore sequencing reads.

FMDV isolate	FMDV P1 amplicon sequence produced by Illumina sequencing (5′ → 3′)
SAT1 KEN 4/98	CAGGCTAAGGATGTCCTTCAGGTACCCCGAGGTAACACGCGACACTCGGGATCTGAGAA<br>GGAGACCAGGAGTTCTATAAAACTGCCTGGTTTAAAAAGCTTCTATGCCTGAATAGGTGA<br>CCGGAGGCCGGCACCTTTTCTTTACCACAAATCCATTTAATGAAGACAACTGACTGTTTTA<br>ACGTTTTACTCGAGATCTTTCACAGGTTCAGACAAACGTTTAAGACAAGCACAGAGATGG<br>AATTCACACTGTACAACGGTGAGAAGAAGACTTTCTACAGCAGACCCAACACACACGGT<br>AACTGCTGGCTCAACTCGCTGCTGCAGCTCTTTCGATACGTCGATGAGCCGCTCTTCGAGG<br>CTGAGTATTTGTCACCAGAGAACAAGACATTGGACATGATCAAACAACTTTCTGATTTCA<br>CTGGACTTGACCTCTCGGATGGTGGGCCACCTGCACTTGTGCTTTGGCTCATCAAGGACT<br>GTCTTTCCACCGGCGTTGGCACCAGCACTCGCCCCAGCGAGATCTGTGTGATCAACGGAG<br>TCGTGATGACACTGGCTGACTTCCATGCTGGCATCTTCATCAAGGGCACCGAACACGCTG<br>TGTTTGCCCTTAACACGTCAGATGGCTGGTACGCCATTGACGATGAAGTGTGCTACCCATG<br>GACACCGGACCCTGCGGACGTACTTGCGTACGTACCGTACGACCAAGAACCCTTGGACG<br>TTGACTGGCAGGACCGGGCTGGCCTGTTCCTACGCGGAGCGGGGCAGTCCTCGCCTGCTA<br>CAGGGTCCCAAAACCAGTCTGGTAACACAGGTAGCATCATCAACAACTACTACATGCAG<br>CAATACCAAAACTCAATGGACACCCAACTTGGAGACAACGCCATTAGTGGTGGCTCAAA<br>TGAAGGGTCTACAGATACCACGTCGACCCACACAAACAACACCCAGAATAATGATTGGT<br>TTTCCAAATTGGCACAATCTGCTTTCAGTGGTCTGGTTGGAGCGCTCTTGGCTGACAAGAA<br>AACGGAAGAAACTACTCTTCTGGAGGATCGCATCCTCACGACAAGCCACGGAACTACCA<br>CCTCCACGACACAGAGCTCGGTCGGCGTGACCTGCGGGTACGCCGAGGCCGACCACTTCC<br>TACCCGGGCCCAACACAAACGGGCTTGAAACACGGGTGGAGCAAGCAGAAAGGTTTTTC<br>AAACACAAACTTTTTGATTGGACCCTCGACCAAAAATTTGGAACAACACACGTTTTGGAA<br>CTGCCCACCGACCACAAAGGCATTTACGGCCAGCTGGTCGACTCCCACTCGTACATCCGT<br>AACGGGTGGGACGTGCAAGTGTCTGCAACTGCCACGCAGTTCAACGGTGGTTGTCTCCTG<br>GTGGCCATGGTACCTGAGCTTTGCAAATTGACAGACAGGGAGAAGTACCAACTCACGCTT<br>TTCCCACACCAATTTCTCAACCCACGGACTAACACCACGGCACACATCCAGGTACCGTAC<br>CTGGGCGTCGACCGGCATGACCAGGGGACACGCCACAAGGCGTGGACGCTGGTTGTGAT<br>GGTGGTTGCTCCATACACAAATGACCAGACAATTGGATCAACAAAGGCTGAGGTCTACGT<br>CAACATCGCACCCACCAACGTGTATGTTGCCGGAGAGAGGCCCGTGAAACAAGGCATCA<br>TCCCTGTGGCCGTCTCCGACGGTTACGGAGGCTTCCAAAACACAGACCCCAAAACATCTG<br>ACCCAATATATGGACACGTACACAACCCCGCGAGGACCGCCTTGCCTGGCAGGTTCACC<br>AACCTGTTGGACGTGGCTGAAGCGTGCCCCACGTTTCTTGACTTCAACGGGGTCCCGTACG<br>TGGCCACCCAGAGCAACTCTGGGAGCAAAGTGCTAGCATGTTTTGATTTGGCTTTTGGAC<br>ACAAAAACCTAAAGAATACCTACATGTCTGGCCTCGCCCAGTACTACACGCAGTACTCGG<br>GCACACTCAATCTGCACTTCATGTACACTGGTCCAACAAACAACAAGGCCAAATACATG<br>GTGGCCTACATCCCACCGGGCACACACCCACTGCCCAACACGCCAGAGATGGCATCGCA<br>CTGCTACCACGCAGAATGGGACACTGGCTTGAATTCAACCTTCACCTTCACAGTGCCGTA<br>CGTGTCAGCGGCGGACTTTGCCTACACCTACTCTGATGAGCCTGAACAGGTTTCAGTCCA<br>GGGTTGGGTGGGTGTCTACCAGATCACGGACACTCACGAGAAGGACGGTGCAGTCGTTGT<br>GACCGTCAGTGCTGGCCCTGACTTTGAGTTCAGGATGCCAATCAGCCCATCCCGCCAGAC<br>CACATCGGCGGGCGAGGGCGCGGATCCAGTGACCACCGACGCTACACAGCACGGTGGTG<br>GACGCCGCACTGCTCGCAGGCACCACACTGATGTCTCGTTCTTACTTGACCGGTTCACCCT

	GGTCGGGAAGACCCAAAACAACAGACTGACACTGGACCTGCTCCAAACCAAGGAGAAA<br>GCACTGGTGGGCGCAATCCTGCGTGCTGCCACGTACTACTTCTCGGATTTGGAGGTGGCGT<br>GTGTTGGCGAGAACAAGTGGGTCGGCTGGACGCCTAATGGCGCGCCAGAGCTCAGTGAA<br>GTCGGCGACAATCCAGTCGTCTTCTCTCACAACGGGACCACCCGCTTTGCTTTGCCTTACA<br>CTGCCCCACACAGGTGTCTTGCTACCGCCTACAACGGCGACTGCAAGTACAAGCCAAATG<br>CTGAGGCACCGCGAACGCACATTCGCGGGGACCTTGCAGTGCTCGCTGAGCGCATCGCCA<br>GTGAGACGCACATCCCAACTACCTTCAATTATGGCAGGATTTACACGGAGGCGGATGTCG<br>ACGTGTACGTGAGAATGAAACGGGCGGAGCTTTACTGCCCCCGTCCGGTGTTAACTCACT<br>ATGACCACAAAGGGCGCGACCGCTACAAAGTGGCCCTGACAAAGCCTGCTAAACAATTG<br>TGCAACTTCGACCTGTTAAAGTTGGCCGGAGACGTTGAGTCCAACCC
SAT2 ZIM 5/81	CAGGCTAAGGATGCCCTTCAGGTACCCCGAGGTAACACGAGACACTCGGGATCTGAGAA<br>GGGGACTAGGAGTTCTATCAAACTGCCTGGTTTAAAAAGCTTCTATGCCTGAATAGGTGA<br>CCGGAGGCCGGCACCTTTTCCTTTTTAACTACTACTATTTAATGGAGACAACTGACTGTTTT<br>AACGTTTTGCTTGAGATCTTCCACAGGTTCAGACAGACGTTCACAGCAGACAGAAAGAT<br>GGAATTCACGCTCTACAACGGAGAGAAGAAGACCTTCTACAGCAGGCCCAACACCCACG<br>GGAACTGTTGGCTCAACTCGCTGTTGCAGCTCTTTCGATACGTCGACGAGCCGCTCTTTGA<br>GTCTGAGTATTTGTCACCTGAAAACAAGACATTGGACATGATCAAACAACTCTCTGATTA<br>CACCAAACTTGACCTTTCAGACGGTGGGCCACCTGCACTCGTGCTTTGGCTCATTAAGGA<br>CTGTCTTCAAACCGGCGTTGGCACTAGCACTCGCCCCAGCGAGATTTGTGTCATCAACGG<br>GGTCGCCATGACCCTGGCTGACTTCCACGCCGGAATATTCATCAAAGGCACCGAACACGC<br>GGTGTTTGCCCTCAACACATCTGAGGGCTGGTACGCCATTGATGATGAGGTGTTCTACCCA<br>TGGACACCCGACCCTGAGAACGTACTCGCGTACGTACCCTACGACCAGGAACCTCTGGA<br>CGTGGATTGGCAGGACCGAGCTGGCCTGTTCCTCCGCGGAGCGGGACAGTCATCACCGGC<br>TACAGGGTCACAGAATCAGTCTGGTAACACTGGAAGCATAATAAACAACTACTACATGC<br>AACAGTACCAGAACTCAATGGACACCCAGCTTGGCGACAACGCCATTTCGGGTGGGTCC<br>AACGAGGGCAGCACGGACACCACGTCCACTCACACCAACAACACTCAAAACAACGATT<br>GGTTTTCCAAATTGGCCCAGTCGGCTATCTCAGGACTCTTCGGAGCCCTGTTGGCTGACAA<br>GAAAACTGAGGAGACCACTCTGCTTGAGGACCGCATCGTGACAACAAGACACGGTACGA<br>CCACCTCCACCACGCAAAGTTCCGTTGGCATCACTTACGGGTATGCTGACGCCGACTCGTT<br>TCGGCCCGGGCCTAACACGTCCGGGTTGGAGACACGTGTCAAACAGGCAGAGCGATTCTT<br>CAAAGAAAAACTTTTTGATTGGACATCAGACAAACCATTCGGCACACTGTACATTTTGGA<br>GTTGCCCAAGGACCACAAAGGGATCTATGGGAGCCTGACTGAGTCTTACGCTTACATGCG<br>TAATGGCTGGGACGTCCAGGTTTCTGCCACGAGCACGCAATTCAACGGCGGTTCGCTTCT<br>CGTGGCCATGGTCCCAGAGCTGTGCTCATTGAAGAGCAGAGAAGAGTTTCAGCTTACTCT<br>GTACCCACACCAGTTCATCAACCCACGGACCAACACCACTGCACACATCCAGGTTCCCTA<br>CCTGGGTGTGAACAGGCACGACCAAGGTAAGCGCCACCAGGCGTGGTCTCTGGTTGTCAT<br>GGTTCTCACGCCTCTTACCACCGAGGCACAGATGAACTCCGGGACAGTTGAGGTATACGC<br>CAACATCGCCCCGACGAACGTGTTCGTTGCTGGCGAAAAGCCCGCAAAGCAAGGCATCA<br>TTCCAGTTGCTTGCTCAGACGGCTATGGCGGGTTCCAAAACACTGACCCGAAGACCGCAG<br>ACCCGATCTACGGTTACGTCTACAACCCGTCTCGGAATGACTGTCACGGTAGGTACTCCA<br>ACCTATTGGACGTCGCCGAGGCGTGTCCCACCCTTTTGAACTTCGACGGGAAACCGTACG<br>TAGTGACAAAGAACAATGGTGATAAAGTTATGACCTGTTTTGACGTGGCATTCACTCACA<br>AGGTACACAAGAACACCTTCCTGGCGGGTCTTGCGGACTACTACACGCAGTACCAAGGCT<br>CATTGAACTACCACTTCATGTACACAGGTCCCACACACCACAAAGCAAAGTTCATGGTGG<br>CGTACATCCCACCGGGTGTGAACACAGACGAACTGCCAAAGACCCCCGAGGCGGCGGCG<br>CACTGCTACCACTCGGAGTGGGACACCGGACTGAACTCCCAGTTCACGTTCGCTGTGCCC<br>TACGTCTCTGCCAGTGACTTCTCCTACACCCACACAGACACGCCCGCAATGGCGACCACC

	AACGGATGGGTGGCGGTTTACCAAGTGACCGACACCCACACGGCGGAGGCAGCCGTTGT<br>TGTGTCTGTGAGCGCCGGACCCGATCTGGAGTTCCGATTCCCGATTGACCCGGTGCGGCA<br>GACCACATCGTCAGGTGAGGGAGCAGACGTAGTCACGACTGATCCCTCCACCCACGGTG<br>GGTCTGTGACGGAGAAGAGGCGCATGCACACCGATGTCGCCTTCGTCATGGACAGGTTCA<br>CCCACGTCCACACCAACCAGACTAGCACAGTGATTGACTTGATGGACACCAATGAGAAG<br>ACCCTTGTGGGTGCGTTGCTCCGTGCTTCCACCTACTACTTCTGTGACATGGAAATTGCTTG<br>CGTTGGCAAACACGCACGCGTGTGGTGGCAACCCAACGGTGCGCCGCGGACAACCCAGC<br>TCCGCGACAACCCAATGGTGTTCTCACACAACAACGTCACACGCTTTGCCCTGCCGTTCA<br>CTGCACCACACCGCCTCCTGTCCACCAGGTACAACGGTGAATGCAAGTACACACAGACA<br>TCAACCGCCATCCGTGGCGACCGTGCTGTGTTGGCAGCCAAATACGCAAATGCAAAGCAT<br>GAGCTTCCCTCCACCTTCAACTTCGGGTACTTGACCGCCGACGAACCAGTCGACGTTTATT<br>ACCGGATGAAGAGGACTGAGCTCTACTGTCCAAGAGCCCTTCTCCCTGCTTATGACCACC<br>AAAGCAGGGACAGGTTCGACGCCCCCATTGGCGTCGAGAAACAACTGTGCAACTTCGAC<br>CTGTTAAAGTTGGCCGGAGACGTTGAGTCCAACCC
SAT2 SAU 1/00	AGGCTAAGGATGCCCTCCAGGTACCCTGAGGTAACAAGCGACACTCAGGATCTGAGGAG<br>GGGACTGGGACTTCTGTAAAAGTGCCCAGTTTAAAAAGCTTCTATGCCTGAATAGGTGAC<br>CGGAGGCCGGCACCTTTTCTTTTTACACAGAAATTACTACATGAACACAACTGATTGTTTT<br>ATCGCTTTGGTAGAAGCTATCAGAGAGATCAAATTTTTGTTTAAACACACCAGAAAGATG<br>GAGTTCACGCTGCACAACGGCGAGAAAAAGACTTTCTACTCAAGGCCCAACCGCCACGA<br>TAACTGCTGGCTAAACACCATCCTGCAATTGTTCAGGTACGTCGATGAGCCATTCTTCGAC<br>TGGGTCTACAATTCACCTGAAAACCTCACGCTCCAGGCAATTGAGCAGCTCGAGGAGCTC<br>ACAGGCCTCAGCCTACACGAGGGTGGGCCCCCCGCTCTCGTGATTTGGAACATCAAACAC<br>TTGCTGCACACCGGAATCGGCACTGCCTCGCGACCCAGCGAGGTGTGCATGGTTGACGGT<br>ACTGACATGTGTCTTGCTGATTTCCACGCAGGAATCTTCCTCAAGGGTGCTGAACACGCCG<br>TGTTCGCCTGTTTGACCTCCAACGGATGGTACGCCATCGACGACGAGGACTTTTACCCATG<br>GACTCCGGATCCGTCCGATGTCTTGTGTTTTGTCCCGTACGACATGGAACCGTTCAACGGA<br>AACGCGATTGCGAAAGCGACCGCGTACGTGAAGGGAGCCGGGCAATCCAGCCCAGCCA<br>CTGGATCGCAAAATCAGTCAGGCAACACTGGTAGCATTATTAACAACTACTACATGCAAC<br>AGTACCAAAACTCGATGGACACACAACTTGGTGACAACGCCATTAGTGGTGGTTCCAACG<br>AGGGGTCGACAGACACTACGTCGACACACACAAACAACACACAGAACAATGATTGGTTC<br>TCCAAGTTGGCCCAATCAGCCATCTCGGGGCTCTTCGGAGCTCTACTGGCCGACAAGAAA<br>ACAGAGGAGACCACACTGTTGGAGGACAGAATTTTGACCACACGTCACGGAACCACGAC<br>CTCCACCACGCAAAGTTCTGTGGGTGTGACACTTGGTTACGCTGATGCTGACTCGTTTCGC<br>CCAGGACCCAACACCTCTGGGCTTGAGACGCGTGTGCAACAGGCAGAACGCTTCTTTAAG<br>GAGAAACTGTTTGACTGGACCAGTGACAAACCTTTCGGCACGCTTTACGTTTTGGAGTTGC<br>CCAAAGACCACAAGGGCATTTACGGTAAACTTACCGACTCCTACACGTACATGCGTAACG<br>GCTGGGACGTACAGGTCAGCGCAACCAGCACACAGTTCAACGGTGGATCACTGCTCGTA<br>GCAATGGTACCAGAGCTGTCTAGTCTGAAAAGTAGAGAAGAATTCCAGCTCACTCTATAC<br>CCACACCAGTTCATCAACCCGCGCACTAACACGACTGCACACATACAGGTCCCGTACCTG<br>GGTGTGAACAGACACGACCAAGGCAAGCGCCACCAGGCGTGGTCTCTGGTTGTGATGGT<br>GCTCACGCCTCTCACCACCGAGGCGCAGATGAACAGCGGCACCGTCGAGGTGTACGCCA<br>ACATCGCACCAACAAATGTAGTTGTGGCGGGTGAGCTGCCAGGCAAACAGGGTATTGTG<br>CCGGTCGCCGCCGCTGACGGGTATGGTGGTTTCCAAAACACCGACCCGAAAACGGCCGA<br>CCCCATTTACGGGTATGTGTACAACCCGTCCAGAAACGACTGCCACGGACGGTTCTCCAA<br>TCTTTTGGATGTCGCCGAGGCGTGTCCAACACTCCTGGATTTTGATGGCAAGCCATATATT<br>GTGACCAAGAACAACGGTGACAAGGTGATGACATCCTTTGACGTCGCCTTCACACACAA<br>GGTGCACAGGAACACGTTTCTGGCGGGCTTGGCTGACTACTACACACAGTACTCAGGCAG

	CCTAAACTACCACTTCATGTACACTGGACCCACACACCACAAGGCAAAGTTCATGGTGGC<br>ATACGTGCCCCCTGGTGTTGAAACTGCACAACTACCTACAACACCGGAGGATGCCGCGCA<br>CTGCTATCACGCGGAATGGGACACTGGACTGAACTCCTCCTTCTCGTTCGCGGTGCCTTAC<br>ATCTCCGCTGCGGACTTCTCCTACACACACACAGACACGCCAGCCATGGCCACCACCAAC<br>GGCTGGGTGATTGTACTGCAGGTCACCGACACGCACTCTGCTGAAGCTGCCGTTGTGGTGT<br>CAGTCAGTGCTGGGCCAGATTTGGAATTTCGGTTCCCTATCGACCCCGTGCGACAGACCA<br>CCTCAGCGGGAGAAAGCGCAGATGTCGTCACCACGGACCCATCTACACACGGTGGAAAC<br>GTGCAAGAGGGCCGACGCAAACACACCGAAGTTGCGTTCCTTCTTGACCGCAGTACACA<br>CGTCCACACAAACAAAACATCCTTTGTTGTGGACCTCATGGACACAAAGGAGAAGGCAC<br>TCGTGGGCGCAATCCTGCGGGCTTCCACCTACTACTTTTGTGACCTTGAGATTGCATGTGT<br>GGGCGACCACACAAGGGCCTTTTGGCAGCCTAACGGGGCGCCGCGGACCACCCAACTTG<br>GCGACAACCCCATGGTTTTCGCCAAGGGCGGTGTGACCCGCTTTGCCATCCCGTTCACGG<br>CCCCACACAGGTTGCTGTCTACTGTCTACAATGGTGAGTGTGTTTACAAGAAAACTCCCAC<br>CGCCATCCGTGGAGACCGTGCGGCGCTAGCGGCAAAGTACGCTGACAGCACGCACACTT<br>TGCCGTCAACCTTCAACTTCGGGTTCGTGACCGTCGACAAACCAGTCGATGTTTACTACCG<br>GATGAAGAGGGCTGAACTGTACTGTCCACGCCCGCTGCTGCCAGCCTATGAACACACAG<br>GCGGAGACAGATTCGACGCGCCCATTGGCGTCGAGAGGCAGACCCTGAACTTCGACCTG<br>TTGAAACAGGCAGGAGACGTTGAGTCCAACCC
SAT3 ZIM 4/81	CAGGCTAAGGATGCCCTTCAGGTACCCCGAGGTAACACGAGACACTCGGGATCTGAGAA<br>GGGGATCGGGAGTTCTTTAAAACTGCCCGGTTTAAAAAGCTTCTATGCCTGAATAGGTGA<br>CCGGAGGCCGGCACCTTTTCCTTTTTATCAACACTAAATTTATGAGGACAACTGACTGTTT<br>TAACGTTCTGCTCGAGATCCTACACAGGTTGAGACAGATTCGCAACACAGGCAGAAAGA<br>TGGAATTCACGCTCTACAACGGAGAGAAGAAGACCTTCTACAGCAGGCCCAACACTCAC<br>GGGAACTGTTGGCTCAACTCACTCCTGCAGCTCTTTCGATACGTCGATGAGCCGCTTTTTG<br>AGTCTGAGTACCTCTCACCTGAAAACAAGACATTGGACATGATCAAACAACTGTCTGATT<br>ACACCAAACTTGACCTTTCAGACGGTGGGCCACCGGCACTCGTGCTCTGGCTCATCAAGG<br>ACTGCCTTCAGACCGGCGTTGGCACCAGTACTCGCCCAAGCGAGATCTGTGTCATCAACG<br>GGGTCGTCATGACCCTGGCTGATTTCCACGCCGGCATTTTCATCAAGGGCACGGAACACG<br>CCGTGTTCGCCCTCAACACATCCGAGGGCTGGTATGCCATTGATGATGAGGTGTTTTATCC<br>ATGGACACCTGACCCTGAGAACGTGCTCGCGTACGTTCCCTACGACCAGGAACCACTGGA<br>CGTTGATTGGCAGGAACGGGCTGGCCTATTCCTCCGCGGAGCAGGCCAATCCTCCCCCGC<br>CACGGGGTCTCAAAATCAATCAGGCAATACTGGTAGCATCATTAACAACTACTACATGCA<br>ACAGTACCAGAACTCCATGGACACCCAGCTTGGCGACAACGCCATCTCGGGTGGATCGA<br>ATGAAGGTAGCACTGACACCACTTCCACCCACACCAACAACACACAGAACAACGACTGG<br>TTCTCAAAGTTGGCGCAGTCTGCCATTTCGGGACTCTTTGGAGCTCTGTTGGCGGACAAGA<br>AGACAGAGGAGACAACTCTTCTGGAGGATCGCATCCTCACCACGCGCCACAACACAACC<br>ACGTCCACCACACAGAGTTCTGTCGGTGTCACATACGGTTACGCGTCAGCTGACCGTTTTC<br>TGCCTGGGCCCAACACCAGTGGACTCGAGACACGCGTTGAACAGGCGGAGAGATTCTTC<br>AAGGAGAAACTCTTCACTTGGACCGCGAGCCAAGAGTACGCACACGTGCATCTACTGGA<br>ACTACCCGTGGACCACAAAGGCATCTACGGTGCCATGCTGGACAGCCACGCATACGTGC<br>GAAATGGCTGGGACGTGCAGGTTTCCGCAACCAGCACACAGTTCAATGGTGGCACTCTCC<br>TCGTCGCCATGGTCCCCGAGCTGAAGACTCTGGACAAGCGTGACGTGTCACAACTCACGC<br>TTTTCCCCCACCAGTTCATCAACCCACGCACCAACACCACCGCACACATCGTGGTACCGT<br>ACGTGGGTGTCAACAGACACGACCAGGCAAAGATGCACAAGGCATGGACACTCGTGGTC<br>GCGGTGCTTGCGCCGCTCACCACGTCAAGCATGGGACAGGACAACGTAGAGGTGTATGC<br>GAACATCGCACCCACCAATGTGTACGTTGCTGGAGAGAAGCCAACAAAACAAGGCATCA<br>TCCCCGTGGCCTGCAACGACGGTTACGGTGGATTCCAGAATACTGACCCAAAGACCGCGG

	ACCCAATCTACGGTCTCGTGTCCAACCCGCCACGCACAGCGTTCCCCGGCAGGTTCACCA<br>ACCTGCTGGATGTCGCCGAAGCGTGCCCAACCTTCCTGGACTTCGACGGCACACCCTACG<br>TCAAGACCAGGCACAACAGCGGGAGCAAAATACTGACACACATTGATTTGGCTTTTGGA<br>CATAAGAGTTTCAAGAACACCTACCTAGCAGGACTCGCCCAGTACTACGCCCAGTACAG<br>CGGGTCTCTGAATCTGCACTTCATGTACACCGGTCCAACGCAGTCTAAAGCACGCTTTATG<br>GTTGCGTACATTCCACCAGGGACCAACCCTGTACCTGACACTCCTGAGGCCGCAGCACAC<br>TGCTACCACTCAGAGTGGGACACCGGACTGAACTCCAAGTTCACGTTTACGGTTCCCTAT<br>ATGTCAGCTGCAGACTTTGCATACACGTACTGTGATGAGCCTGAACAGGCTTCAGCACAG<br>GGGTGGGTGACACTCTACCAAATTACAGACACGCACGACCCCGATTCGGCGGTGCTTGTC<br>TCGGTCAGTGCTGGCGCTGACCTTGAATTTCGGCTCCCAATCAACCCTGCAACCCAGACA<br>ACCAGCTCAGGTGAAGGTGGTGATGTTGTGACGACCGACGTCACGACACACGGTGGAAC<br>CGTGGACCAACCACGCCGCCAACACACCAACGTGGAGTTTCTGCTGGACAGATTCACAC<br>ACATTGGTGAGATCACCGCCTCCAAGACAATTGACCTCCTGGACACGAAGGAACACACG<br>CTGGTGGGCGCAATCCTGCGTTCTGCCACGTATTACTTCTGTGATCTAGAGGTTGCCGTCC<br>TGGGCACAAGCCAGTGGGTGGCGTGGGTTCCCAACGGATGCCCACACACCGACCGCGTG<br>GAGGACAACCCAGTCGTTCACGCGAAGAACGGTGTCACCCGCTTCGCTCTGCCGTACACA<br>GCACCACACCAGGTTCTCGCTACCGTGTACAACGGCAACTGCAAATATTCCAACACCCAA<br>CACGTTACGCCACGCCGTGGTGACATGGCCGTGTTAGCACAACGTGTTGCGAATGAAACC<br>ACAAGATGCACGCCCACAACTTTCAACTTCGGGAGATTGTTGTGTGACACTGGTTCGGTTT<br>ACTACCGGATGAAGAGAGCTGAGCTGTACTGCCCACGGCCCCTCATGGTCAGGTACACGC<br>ACACCACTGACCGGTACAAAATCAAACTGGTCGCACCTGACAAACAATTGTGCAACTTCG<br>ACCTGTTAAAGTTGGCCGGAGACGTTGAGTCCAACCC

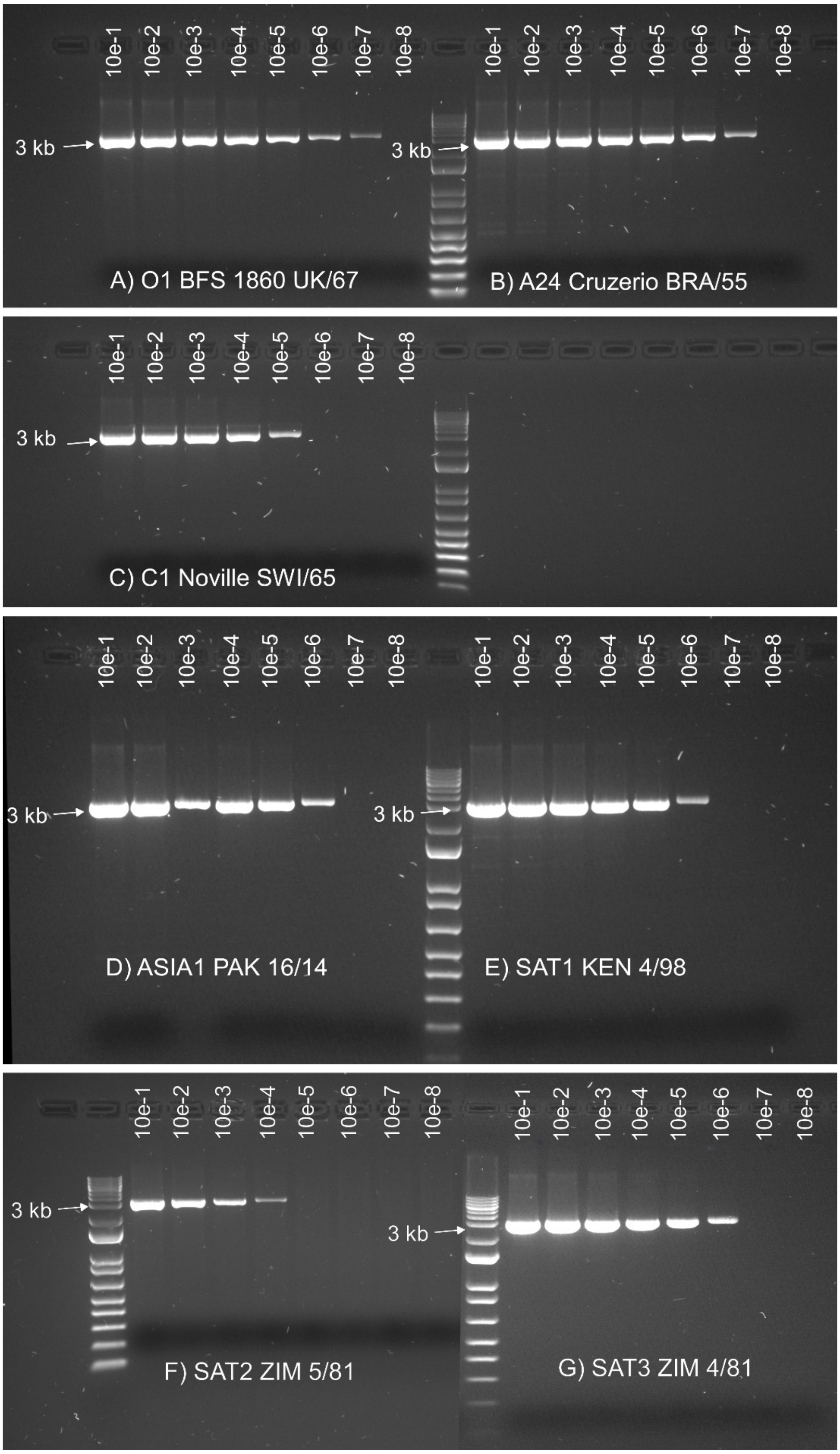
A) O1 BFS 1860 UK/67
B) A24 Cruzerio BRA/55
C) C1 Noville SWI/65
D) ASIA1 PAK 16/14
E) SAT1 KEN 4/98
F) SAT2 ZIM 5/81
G) SAT3 ZIM 4/81
3 kb
10e-1
10e-2
10e-3
10e-4
10e-5
10e-6
10e-7
10e-8

Figure S1. A 1% agarose gel demonstrating P1 amplification of 10-fold diluted representative FMDV cell culture isolates. Serotypes are as follows: A) O1 BFS 1860 UK/67, B) A24 Cruzerio BRA/55, C) C1 Noville SWI/65, D) ASIA1 PAK 16/14, E) SAT1 KEN 4/98, F) SAT2 ZIM 5/81, G) SAT3 ZIM 4/81.

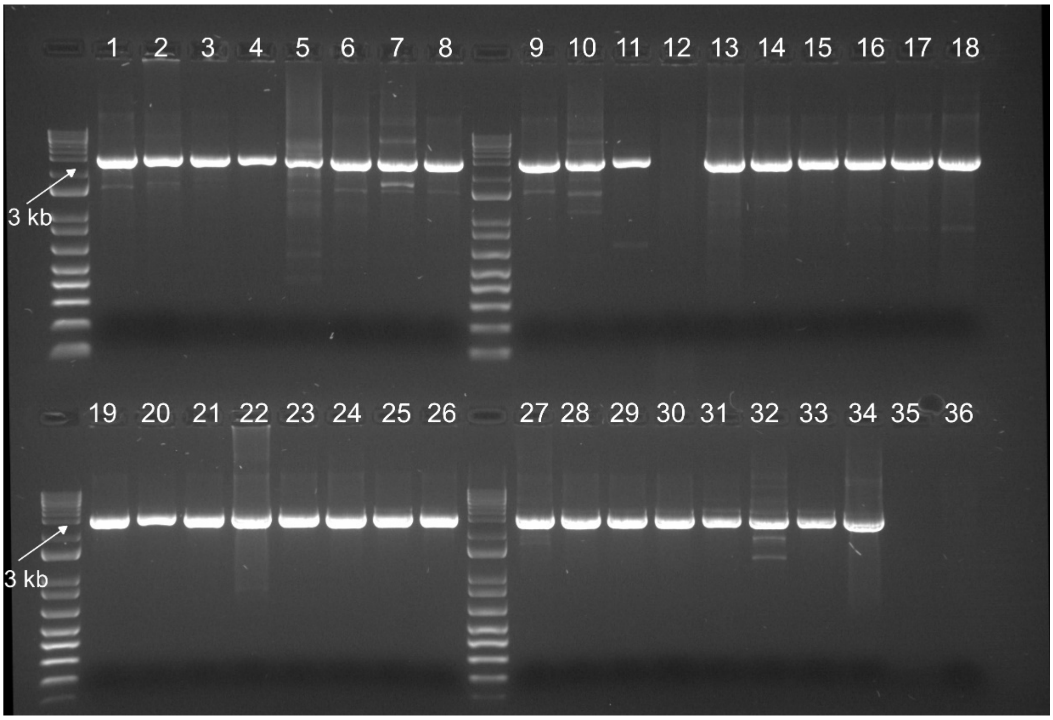

Figure S2. A 1% agarose gel demonstrating P1 amplification of FMDV matrices samples from experimentally infected animals. Sample numbers are as follows:

Sample No.	Sample Name	Sample No.	Sample Name
1	C1325 milk 4 dpi	19	P1–6 GRP A OF 3 dpi
2	C1418 milk 4 dpi	20	P7–12 GRP B OF 3 dpi
3	C1511 milk 4 dpi	21	P13–18 GRP C OF 3 dpi
4	C1520 milk 4 dpi	22	C1924 ves.fluid 4 dpi
5	C1325 serum 4 dpi	23	P252 o.swab 2 dpi
6	C1520 serum 4 dpi	24	P187 o.swab 2 dpi
7	C1520 o.swab 3 dpi	25	P168 Tonsil 7 dpi
8	C1520 o.swab 6 dpi	26	S21 n.swab 4 dpi
9	C1511 n.swab 2 dpi	27	S23 n.swab 4 dpi
10	C1418 n.swab 4 dpi	28	P GRP OF 3 dpi
11	P247 o.swab 5 dpi	29	P GRP OF 4 dpi
12	P257 Submand.LN 6 dpi	30	P GRP OF 5 dpi
13	P3 tissue 3 dpi	31	P78 Interdigital 4 dpi
14	C1727 serum 4 dpi	32	P206 o.swab 5 dpi
15	P13 serum 3 dpi	33	P204 Prescap.LN 7 dpi
16	C1722 n.swab 4 dpi	34	P209 P217 P222 combined VF 4 dpi

17	C1750 n.swab 4 dpi	35	PBS-1
18	P13 epi.lesion 3 dpi	36	PBS-2

Note: Animal species for each sample is indicated by "C" for cattle, "P" for pigs, and "S" for sheep. SM LN = submandibular lymph node, ves lesion = vesicular lesion, and prescap LN = prescapular lymph node.

Table S2. Mean read depth and coverage of various 10-fold diluted FMDV cell culture isolates. Minimum read depth cutoff used is 50X or 1% of the mean read depth if the value is greater than 50.

Sample	Reference	Mean Read Depth	Depth cutoff (1% of mean read depth)	% Coverage at depth cutoff or greater
O1 BFS 10e-1	AY593815.1	12,414.99	124.15	99.44
O1 BFS 10e-2	AY593815.1	19,117.24	191.17	99.30
O1 BFS 10e-3	AY593815.1	9,559.87	95.60	99.47
O1 BFS 10e-4	AY593815.1	17,994.96	179.95	99.47
O1 BFS 10e-5	AY593815.1	6,493.13	64.93	99.54
O1 BFS 10e-6	AY593815.1	5,431.53	54.32	99.37
O1 BFS 10e-7	AY593815.1	3,149.82	50.00	99.83
O1 BFS 10e-8	AY593815.1	1.87	50.00	0.00
A24 10e-1	AY593768.1	15,934.44	159.34	99.31
A24 10e-2	AY593768.1	4,229.17	50.00	99.64
A24 10e-3	AY593768.1	12,393.05	123.93	99.40
A24 10e-4	AY593768.1	3,533.36	50.00	99.54
A24 10e-5	AY593768.1	9,224.74	92.25	99.50
A24 10e-6	AY593768.1	7,512.79	75.13	99.37
A24 10e-7	AY593768.1	3,248.42	50.00	99.64
A24 10e-8	AY593768.1	12.75	50.00	0.00
C 10e-1	AJ133357.1	33,071.34	330.71	99.33
C 10e-2	AJ133357.1	51,766.85	517.67	99.27
C 10e-3	AJ133357.1	26,055.92	260.56	99.43
C 10e-4	AJ133357.1	43,821.35	438.21	99.33
C 10e-5	AJ133357.1	17,960.59	179.61	99.43
C 10e-6	AJ133357.1	19.43	50.00	0.00
C 10e-7	AJ133357.1	23.75	50.00	0.00
C 10e-8	AJ133357.1	19.00	50.00	0.00
ASIA1 10e-1	AY593795.1	26,763.52	267.64	99.30
ASIA1 10e-2	AY593795.1	14,635.04	146.35	99.30
ASIA1 10e-3	AY593795.1	23,121.93	231.22	99.43
ASIA1 10e-4	AY593795.1	11,868.71	118.69	99.47
ASIA1 10e-5	AY593795.1	18,235.64	182.36	99.34
ASIA1 10e-6	AY593795.1	8,006.40	80.06	99.67
ASIA1 10e-7	AY593795.1	2.80	50.00	0.00
ASIA1 10e-8	AY593795.1	4.28	50.00	0.00
SAT1 10e-1	09_SAT1_Ken_consensus	20,463.33	204.63	99.60

SAT1 10e-2	09_SAT1_Ken_consensus	23,686.28	236.86	99.60
SAT1 10e-3	09_SAT1_Ken_consensus	24,045.51	240.46	99.70
SAT1 10e-4	09_SAT1_Ken_consensus	18,105.97	181.06	99.67
SAT1 10e-5	09_SAT1_Ken_consensus	15,482.83	154.83	99.64
SAT1 10e-6	09_SAT1_Ken_consensus	6,560.87	65.61	99.64
SAT1 10e-7	AY593795.1	8.60	50.00	0.00
SAT1 10e-8	AY593795.1	5.11	50.00	0.00
SAT2 10e-1	10_SAT2_Zim_consensus	18,341.56	183.42	99.60
SAT2 10e-2	10_SAT2_Zim_consensus	11,568.53	115.69	99.60
SAT2 10e-3	10_SAT2_Zim_consensus	15,781.76	157.82	99.60
SAT2 10e-4	10_SAT2_Zim_consensus	1,607.99	50.00	99.77
SAT2 10e-5	12_SAT3_Zim_consensus	2.24	50.00	0.00
SAT2 10e-6	AY593768.1	1.96	50.00	0.00
SAT2 10e-7	12_SAT3_Zim_consensus	4.98	50.00	0.00
SAT2 10e-8	10_SAT2_Zim_consensus	14.44	50.00	0.00
SAT3 10e-1	12_SAT3_Zim_consensus	19,359.73	193.60	99.60
SAT3 10e-2	12_SAT3_Zim_consensus	25,179.56	251.80	99.60
SAT3 10e-3	12_SAT3_Zim_consensus	18,578.82	185.79	99.64
SAT3 10e-4	12_SAT3_Zim_consensus	22,526.20	225.26	99.60
SAT3 10e-5	12_SAT3_Zim_consensus	12,205.67	122.06	99.60
SAT3 10e-6	12_SAT3_Zim_consensus	10,564.80	105.65	99.77
SAT3 10e-7	12_SAT3_Zim_consensus	8.69	50.00	0.00
SAT3 10e-8	12_SAT3_Zim_consensus	7.84	50.00	0.00

Table S3. Mean read depth and coverage of various clinical FMDV samples from experimentally infected animals. Minimum read depth cutoff used is 50X or 1% of the mean read depth if the value is greater than 50.

Sample	Reference	Mean Read Depth	Depth cutoff (1% of mean read depth)	% Coverage at depth cutoff or greater
C1325 milk 4 dpi	AY593795.1	5,518.48	55.18	99.37
C1418 milk 4 dpi	AY593795.1	4,261.95	50.00	99.40
C1511 milk 4 dpi	AY593795.1	2,522.99	50.00	99.57
C1520 milk 4 dpi	AY593795.1	6,054.23	60.54	99.53
C1325 serum 4 dpi	KY091304.1	2,306.98	50.00	100.00
C1520 serum 4 dpi	AY593795.1	6,516.59	65.17	99.30
C1520 o.swab 3 dpi	AY593795.1	3,371.82	50.00	99.40
C1520 o.swab 6 dpi	AY593795.1	3,028.93	50.00	99.53
C1511 n.swab 2 dpi	AY593795.1	5,951.86	59.52	99.27
C1418 n.swab 4 dpi	AY593795.1	2,576.51	50.00	99.40
P247 o.swab 5 dpi	JF739177.1	13,259.32	132.59	99.57
P257 Submand.LN 6 dpi	JF739177.1	2.01	50.00	0.00
P3 tissue	JF739177.1	6,769.89	67.70	99.60
P3 tissue 3 dpi	AJ539141.1	25,988.01	259.88	99.27

C1727 serum 4 dpi	AJ539141.1	38,713.99	387.14	99.27
P13 serum 3 dpi	AJ539141.1	16,001.16	160.01	99.40
C1722 n.swab 4 dpi	AJ539141.1	28,029.02	280.29	99.27
C1750 n.swab 4 dpi	AJ539141.1	12,912.59	129.13	99.27
P13 epi.lesion 3 dpi	AJ539141.1	20,790.84	207.91	99.34
P1–6 GRP A OF 3 dpi	AJ539141.1	16,443.03	164.43	99.27
P7–12 GRP B OF 3 dpi	AJ539141.1	11,192.16	111.92	99.37
P13–18 GRP C OF 3 dpi	AJ539141.1	7,013.01	70.13	99.64
C1924 ves.fluid 4 dpi	AY593823.1	23,427.82	234.28	99.34
P252 o.swab 2 dpi	AY593763.1	13,508.21	135.08	99.31
P187 o.swab 2 dpi	AY593763.1	17,325.97	173.26	99.34
P168 Tonsil 7 dpi	AY593763.1	6,635.34	66.35	99.47
S21 n.swab 4 dpi	AY593763.1	7,699.39	76.99	99.64
S23 n.swab 4 dpi	AY593763.1	30,379.84	303.80	99.31
P GRP OF 3 dpi	AY593763.1	16,398.15	163.98	99.31
P GRP OF 4 dpi	AY593763.1	21,176.20	211.76	99.34
P GRP OF 5 dpi	AY593763.1	5,428.62	54.29	99.80
P78 Interdigital 4 dpi	10_SAT2_Zim_consensus	10,005.49	100.05	99.60
P206 o.swab 5 dpi	10_SAT2_Zim_consensus	7,879.73	78.80	99.64
P204 Prescap.LN 7 dpi	10_SAT2_Zim_consensus	6,850.83	68.51	99.83

Note: Animal species for each sample is indicated by “C” for cattle, “P” for pigs, and “S” for sheep. SM LN = submandibular lymph node, ves lesion = vesicular lesion, and prescap LN = prescapular lymph node.

Table S4. Mean read depth of various FMDV isolates sequenced via Nanopore (flongle flow cell) and Illumina (nano flow cell) sequencing.

Sample	Nanopore Mean Read Depth	Illumina Mean Read Depth
O TUR 1/69	14,932.43	6,565.90
O UKG 1/67	14,129.53	7,631.70
A BRA 1/55	13,695.27	5,816.30
A IRQ 24/64	21,941.99	14,445.90
C SWI 1/65	20,506.09	8,803.50
ASIA1 ISR 1/89	17,835.41	5,500.90
ASIA1 PAK 1/54	16,673.27	7,851.30
SAT1 KEN 4/98	15,345.97	6,076.70
SAT2 ZIM 5/81	7,959.86	18,897.70
SAT2 SAU 1/00	2,480.68	5,552.80
SAT3 ZIM 4/81	17,665.61	8,936.30

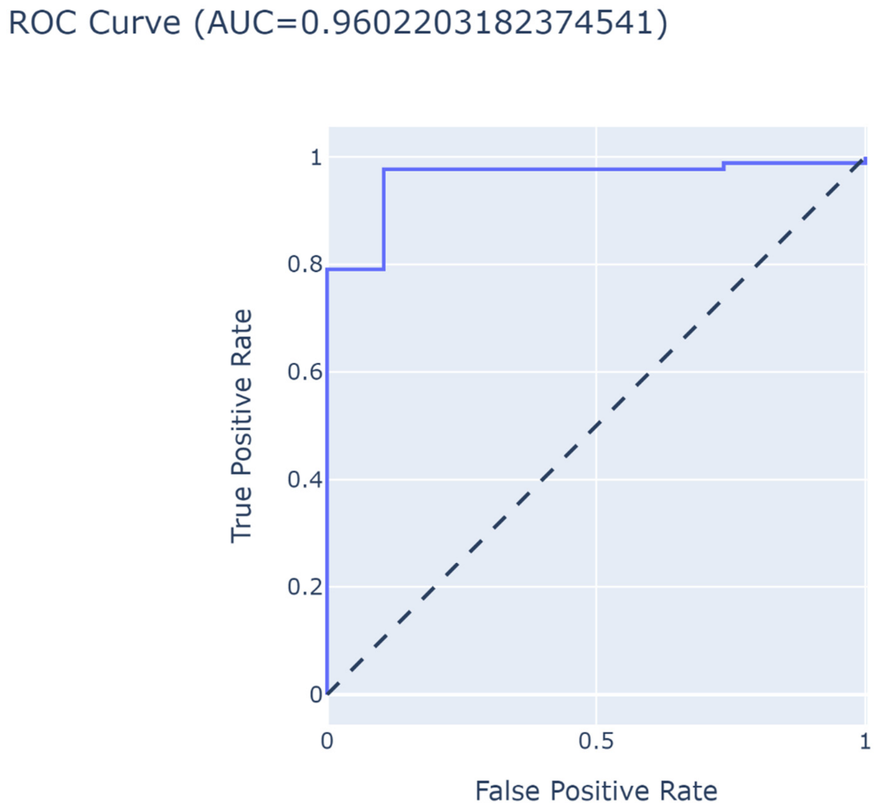

Figure S3. Receiver Operating Characteristic (ROC) curve using the RT-qPCR as the determiner for positive or negative samples to measure false positive rate and true positive rate of the inverse ratio of unmapped reads to total reads generated by the FMDV-ONTAPS protocol. Area Under the Curve (AUC) value is 0.96 and indicates a good fitting model.

Table S5. FMDV-ONTAPS protocol thresholds for each False Positive Rate (FPR) and True Positive Rate (TPR).

False Positive Rate	True Positive Rate	Threshold
0	0	0.321767
0	0.011628	0.327324
0	0.790698	0.332228
0.105263	0.790698	0.3323
0.105263	0.976744	0.646498
0.736842	0.976744	0.927454
0.736842	0.988372	0.927871
1	0.988372	0.942086
1	1	1

Table S6. PCR positive (1) or negative (0), number of unmapped reads, total number of reads, and the inverse proportion of unmapped to total reads for each sample evaluated with the FMDV-ONTAPS protocol.

Samples	PCR Positive (1) or Negative (0)	Unmapped Reads	Total Reads	Inverse Percent Unmapped Reads
O TUR 1/69	1	762	40,447	0.981160531
O UKG 1/67	1	557	40,125	0.98611838
A BRA 1/55	1	1,010	38,425	0.973715029
A IRQ 24/64	1	1,184	64,603	0.981672678
C SWI 1/65	1	794	54,724	0.985490827
ASIA1 ISR 1/89	1	909	51,684	0.982412352
ASIA1 PAK 1/54	1	1,013	43,701	0.976819752
SAT1 KEN 4/98	1	421	41,014	0.989735212
SAT2 ZIM 5/81	1	2,049	27,598	0.92575549
SAT2 SAU 1/00	1	4,579	10,840	0.577583026
SAT3 ZIM 4/81	1	1,466	46,804	0.968677891
PBS	0	1,056	1,153	0.084128361
O_10e-1	1	606	32,518	0.981364168
O_10e-2	1	575	60,971	0.990569287
O_10e-3	1	2,192	28,670	0.923543774
O_10e-4	1	501	36,389	0.986232103
O_10e-5	1	446	26,110	0.982918422
O_10e-6	1	358	13,940	0.974318508
O_10e-7	1	522	1,372	0.619533528
O_10e-8	0	831	850	0.022352941
A_10e-1	1	964	45,813	0.978957938
A_10e-2	1	308	34,929	0.991182112
A_10e-3	1	638	50,613	0.987394543
A_10e-4	1	203	31,623	0.993580622
A_10e-5	1	374	24,750	0.984888889
A_10e-6	1	462	11,307	0.959140356
A_10e-7	0	1,213	1,977	0.386444107
A_10e-8	0	698	731	0.045143639
C_10e-1	1	2,123	90,606	0.976568881
C_10e-2	1	1,282	135,661	0.990549974
C_10e-3	1	3,384	68,882	0.950872507
C_10e-4	1	1,065	109,167	0.990244305
C_10e-5	1	1,023	45,572	0.977552006
C_10e-6	0	1,131	1,349	0.161601186

C_10e-7	0	2,312	2,371	0.024884015
C_10e-8	0	2,256	2,351	0.040408337
ASIA1_10e-1	1	1,619	79,111	0.979535084
ASIA1_10e-2	1	455	43,568	0.989556555
ASIA1_10e-3	1	1,101	62,663	0.982429823
ASIA1_10e-4	1	280	36,575	0.992344498
ASIA1_10e-5	1	776	48,813	0.984102596
ASIA1_10e-6	1	481	21,326	0.977445372
ASIA1_10e-7	0	548	561	0.023172906
ASIA1_10e-8	0	870	897	0.030100334
SAT1_10e-1	1	963	56,623	0.982992777
SAT1_10e-2	1	992	67,040	0.985202864
SAT1_10e-3	1	910	63,449	0.985657772
SAT1_10e-4	1	437	47,840	0.990865385
SAT1_10e-5	1	2,060	42,179	0.95116053
SAT1_10e-6	0	1,308	17,832	0.926648721
SAT1_10e-7	0	1,791	1,825	0.018630137
SAT1_10e-8	0	545	569	0.042179262
SAT2_10e-1	1	1,298	65,623	0.98022035
SAT2_10e-2	1	546	41,909	0.986971772
SAT2_10e-3	1	781	51,615	0.98486874
SAT2_10e-4	1	287	6,861	0.958169363
SAT2_10e-5	1	472	484	0.024793388
SAT2_10e-6	1	330	346	0.046242775
SAT2_10e-7	0	653	678	0.036873156
SAT2_10e-8	0	1,052	1,113	0.054806828
SAT3_10e-1	1	897	53,095	0.983105754
SAT3_10e-2	1	1,195	73,982	0.983847422
SAT3_10e-3	1	989	48,405	0.979568226
SAT3_10e-4	1	595	61,555	0.990333848
SAT3_10e-5	1	2,138	34,680	0.938350634
SAT3_10e-6	1	978	28,586	0.965787448
SAT3_10e-7	0	1,503	1,541	0.024659312
SAT3_10e-8	0	1,158	1,184	0.021959459
C1325_milk_4dpi	1	5,657	41,380	0.863291445
C1418_milk_4dpi	1	20,353	48,822	0.583118266
C1511_milk_4dpi	1	2,450	16,654	0.852888195
C1520_milk_4dpi	1	4,816	44,735	0.892343802
C1325_sera_4dpi	1	1,138	17,368	0.934477199
C1520_sera_4dpi	1	1,530	41,217	0.962879394
C1520_oswab_3dpi	1	1,442	22,880	0.936975524

C1520_oswab_6dpi	1	1,563	21,822	0.928375034
C1511_nswab_2dpi	1	2,821	42,168	0.93310093
C1418_nswab_4dpi	1	1,173	20,154	0.941798154
P247_oswab_5dpi	1	767	38,262	0.979954001
P257_SM_LN_6dpi	1	853	863	0.011587486
P3_tissue_3dpi	1	4,611	29,148	0.841807328
C1727_sera_4dpi	1	200	20,922	0.990440684
P13_sera_3dpi	1	390	26,118	0.985067769
C1722_nswab_4dpi	1	725	36,890	0.980346978
C1750_nswab_4dpi	1	516	20,966	0.975388725
P13_epi_lesion_3dpi	1	1,494	30,862	0.951590953
P1–6_OF_grp_A_3dpi	1	485	17,697	0.972594225
P7–12_OF_grp_B_3dpi	1	487	29,389	0.983429174
P13–18_OF_grp_C_3dpi	1	1,361	68,654	0.980175955
C1924_VF_4dpi	1	24,662	45,924	0.462982319
P252_oswab_2dpi	1	2,219	70,522	0.968534642
P187_oswab_5dpi	1	411	43,044	0.990451631
P168_tonsil_7dpi	1	3,441	50,850	0.932330383
S21_nswab_4dpi	1	5,459	32,003	0.829422242
S23_nswab_4dpi	1	11,348	44,888	0.747193014
P_GRP_OF_3dpi	1	1,052	86,802	0.987880464
P_GRP_OF_4dpi	1	870	45,218	0.980759874
P_GRP_OF_5dpi	1	1,244	62,641	0.980140802
P78_interdigital_4dpi	1	3,883	29,605	0.868839723
P206_oswab_5dpi	1	1,258	36,688	0.965710859
P204_PS_LN_7dpi	1	3,596	28,097	0.872014806
P209-217-222_combined_VF_4dpi	1	6,115	29,545	0.793027585
PBS-1	0	648	674	0.038575668
PBS-2	0	105	116	0.094827586

Note: Animal species for each sample is indicated by “C” for cattle, “P” for pigs, and “S” for sheep. SM LN = submandibular lymph node, ves lesion = vesicular lesion, and prescap LN = prescapular lymph node.

Table S7. Cost per sample in USD, turnaround time, sensitivity, and equipment cost comparisons in USD between Sanger, Illumina, and Nanopore Sequencing methods.

Assay	Cost per sample	Turnaround time	Sensitivity	Equipment Cost
Sanger Sequencing	$22.54	12 hours	10^{-5} to 10^{-7}	$310,000
Illumina Sequencing	$51.27	17 hours	10^{-5} to 10^{-7}	$72,033
Nanopore Sequencing	$32.91	7 hours	10^{-5} to 10^{-7}	$4,761

Supplemental Protocol: Bash analysis commands used to produce results for the FMDV-ONTAPS protocol:

```
#!/usr/bin/env bash

###=============================================
### 1. Set variables and combine fastq.gz files
###=============================================

REFERENCE=/mnt/d/references/FMD_prototype_sequences.fasta #choose your desired reference file
RUN_DIR=/mnt/d/nanopore_rundata/runfolder/20250607*** #choose your sequencing run folder
ANALYSIS_DIR=$RUN_DIR/..

mkdir -p $ANALYSIS_DIR/analysis/01_minimap2
mkdir -p $ANALYSIS_DIR/analysis/02_bam
mkdir -p $ANALYSIS_DIR/analysis/03_sorted_bam
mkdir -p $ANALYSIS_DIR/analysis/04_mapping_summary

MINIMAP2_DIR=$ANALYSIS_DIR/analysis/01_minimap2
SAMTOOLS_DIR=$ANALYSIS_DIR/analysis/02_bam
SORTEDBAM_DIR=$ANALYSIS_DIR/analysis/03_sorted_bam
SUMMARY_DIR=$ANALYSIS_DIR/analysis/04_mapping_summary

for f in $RUN_DIR/fastq_pass/barcode**
   barcode=${f##*/}
   cat ${f}/*.fastq.gz >> ${f}/combined_fastq_pass/${barcode}_combined.fastq.gz
done

###=============================================
### 2. Minimap2 (map reads to reference sequences)
###=============================================

conda activate minimap2-2.28 #create conda environment with minimap2 installed

cd $ANALYSIS_DIR

for f in $RUN_DIR/fastq_pass/barcode**
   barcode=${f##*/}
   minimap2 -ax map-ont $REFERENCE ${f}/combined_fastq_pass/*.fastq.gz >
   $MINIMAP2_DIR/${barcode}.sam -t 8
done

conda deactivate
```

```
###=================================================
### 3. Samtools (convert sam to bam, sort bam file, create index, produce consensus sequence, and create
summary statistics)
###=================================================

conda activate samtools-1.21 #create conda environment with samtools installed

for f in $MINIMAP2_DIR/barcode**.sam
  barcode=${f%.*}
samtools view -S --threads 8 -F 2304 --reference $REFERENCE -bS ${barcode}.sam >
  $SAMTOOLS_DIR/${barcode}.bam
samtools sort -m 2G $SAMTOOLS_DIR/${barcode}.bam -o $SORTEDBAM_DIR/${barcode}_sorted.bam
  samtools index $SORTEDBAM_DIR/${barcode}_sorted.bam
  mkdir -p $SUMMARY_DIR/${barcode}_output
samtools flagstat $SORTEDBAM_DIR/${barcode}_sorted.bam >
  $SUMMARY_DIR/${barcode}_output/${barcode}_flagstats.tsv
samtools idxstats $SORTEDBAM_DIR/${barcode}_sorted.bam | sort -k 3 -r -n >
  $SUMMARY_DIR/${barcode}_output/${barcode}_idxstats.tsv
ACCESSION_NUMBER=$(awk 'NR==1 {print $1}'
  $SUMMARY_DIR/${barcode}_output/${barcode}_idxstats.tsv)
samtools consensus -m bayesian -f fasta -a -A -d 10 --het-scale 0.55 -r $ACCESSION_NUMBER
  $SORTEDBAM_DIR/${barcode}_sorted.bam -o
  $SUMMARY_DIR/${barcode}_output/${barcode}_consensus.fasta
samtools depth -a $SORTEDBAM_DIR/${barcode}_sorted.bam >
  $SUMMARY_DIR/${barcode}_output/${barcode}_depth.tsv
awk -v an="$ACCESSION_NUMBER" '($1 == an)'
  $SUMMARY_DIR/${barcode}_output/${barcode}_depth.tsv >
  $SUMMARY_DIR/${barcode}_output/${barcode}_depth_filtered.tsv
done
```
